# Regulation of Dense-Core Granule Replenishment by Autocrine BMP Signalling in *Drosophila* Secondary Cells

**DOI:** 10.1371/journal.pgen.1006366

**Published:** 2016-10-11

**Authors:** Siamak Redhai, Josephine E. E. U. Hellberg, Mark Wainwright, Sumeth W. Perera, Felix Castellanos, Benjamin Kroeger, Carina Gandy, Aaron Leiblich, Laura Corrigan, Thomas Hilton, Benjamin Patel, Shih-Jung Fan, Freddie Hamdy, Deborah C. I. Goberdhan, Clive Wilson

**Affiliations:** 1 Department of Physiology, Anatomy and Genetics, University of Oxford, Oxford, United Kingdom; 2 Nuffield Department of Surgical Sciences, University of Oxford, John Radcliffe Hospital, Oxford, United Kingdom; Washington University in Saint Louis School of Medicine, UNITED STATES

## Abstract

Regulated secretion by glands and neurons involves release of signalling molecules and enzymes selectively concentrated in dense-core granules (DCGs). Although we understand how many secretagogues stimulate DCG release, how DCG biogenesis is then accelerated to replenish the DCG pool remains poorly characterised. Here we demonstrate that each prostate-like secondary cell (SC) in the paired adult *Drosophila melanogaster* male accessory glands contains approximately ten large DCGs, which are loaded with the Bone Morphogenetic Protein (BMP) ligand Decapentaplegic (Dpp). These DCGs can be marked in living tissue by a glycophosphatidylinositol (GPI) lipid-anchored form of GFP. In virgin males, BMP signalling is sporadically activated by constitutive DCG secretion. Upon mating, approximately four DCGs are typically released immediately, increasing BMP signalling, primarily via an autocrine mechanism. Using inducible knockdown specifically in adult SCs, we show that secretion requires the Soluble NSF Attachment Protein, SNAP24. Furthermore, mating-dependent BMP signalling not only promotes cell growth, but is also necessary to accelerate biogenesis of new DCGs, restoring DCG number within 24 h. Our analysis therefore reveals an autocrine BMP-mediated feedback mechanism for matching DCG release to replenishment as secretion rates fluctuate, and might explain why in other disease-relevant systems, like pancreatic β-cells, BMP signalling is also implicated in the control of secretion.

## Author Summary

Glands and neurons concentrate and store the signals that they secrete in dense-core granules (DCGs). They are released in response to the appropriate stimulus in a regulated fashion. Since release rate varies with the level of stimulation, it must be matched to DCG biogenesis to replenish the DCG pool rapidly after secretion. However, the mechanisms by which DCG release is sensed and then co-ordinated with biogenesis are not understood. Here we genetically dissect this process in the prostate-like secondary cells of the adult male *Drosophila* accessory gland. These cells form large DCGs that are secreted into seminal fluid. We find that these DCGs are loaded with a specific signalling molecule, the Bone Morphogenetic Protein (BMP), Decapentaplegic (Dpp). When males mate, a number of DCGs are rapidly secreted, stimulating BMP signalling primarily via an autocrine mechanism. This BMP signal accelerates DCG biogenesis, so that DCG number is fully replenished within 24 h. Our work therefore suggests that at least in secondary cells, BMP signalling plays a simple autocrine role in matching DCG release to new biogenesis. Interestingly, BMP signalling affects secretion in other cell types, such as pancreatic β-cells, suggesting possible evolutionary conservation of this homeostatic mechanism.

## Introduction

Regulated secretion of neurotransmitters, hormones and enzymes from neuronal, endocrine and exocrine cells typically involves fusion of specialised secretory compartments with the plasma membrane. Prior to secretion, the bioactive molecules are packaged into dense-core granules (DCGs), which form via a series of membrane trafficking and maturation events [1,2]. Cargos are first clustered together at the trans-Golgi network, and bud off in membrane-bound compartments. They are then sorted from non-DCG proteins by specific interactions with adaptor molecules [2] and condense together in a maturation process that requires intraluminal Ca^2+^ ions and acidification [3,4].

By studying DCG biogenesis in cultured mammalian secretory cells, some key regulators have been identified. For example, intraluminal proteins called granins complex with cargos and drive formation and budding off of immature DCGs from the Golgi [5,6]. Cargo clustering at the trans-Golgi network also involves cholesterol and lipid raft-like structures [7], and requires the activity of specific enzymes associated with lipid metabolism [8,1].

When stimulated by secretagogues, mature DCGs fuse to the plasma membrane in a Ca^2+^-dependent fashion via a process involving synaptotagmins, SNAP (Soluble NSF Attachment Protein) Receptors (SNAREs) and vesicle-associated membrane proteins (VAMPs) [9,10]. Secretory cells must increase DCG biogenesis to rapidly replenish released DCGs. There is good evidence for elevated transcription and translation of mRNAs encoding DCG proteins after secretion [11]. But the mechanisms by which either these processes or membrane trafficking itself are modulated in response to secretion are unknown, despite their likely importance in maintaining secretion rates in several disease-relevant systems, particularly pancreatic β-cells [1]. A number of signalling molecules have been implicated in regulating secretion. For example, insulin release from β-cells is affected by Hedgehog proteins [12], Wnts [13] and BMPs [14]. However, models to genetically dissect out DCG biogenesis control *in vivo* are very limited, and none of these signals has yet been implicated in triggering elevated DCG biogenesis in response to exocytosis.

**Fig 1:**
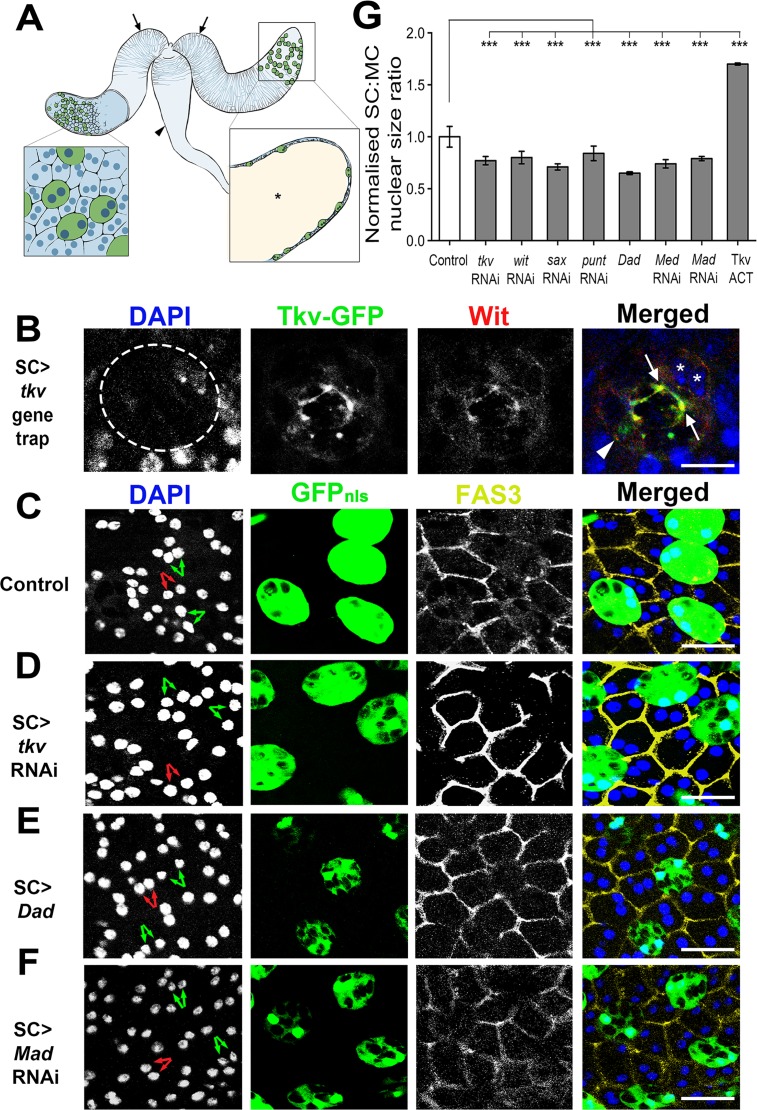
Multiple BMP signalling components regulate nuclear growth in SCs. **A.** Schematic of the paired male accessory glands (arrows), which pump their contents into the ejaculatory duct (arrowhead) during mating. Left inset shows the epithelial secretory monolayer containing secondary cells (SCs; green) and main cells (MCs), all of which are binucleate. Right inset is a section through the gland revealing the large lumen (asterisk). **B.** SC (circled) expressing a gene trap for the BMP type I receptor *tkv*, and stained with an antibody against the BMP type II receptor Wit. These receptors are present both on the plasma membrane (arrowhead) and co-localise in intracellular compartments (arrow). DAPI marks nuclei blue in SCs (asterisks) and surrounding non-expressing MCs. **C-F.** Accessory glands (AGs) from 6-day-old controls (C) and males expressing RNAis targeting *tkv* (D) or *Mad* (F) or expressing the BMP signalling antagonist *Dad* (E) in adult SCs under the control of esgF/O^ts^ after temperature shift at eclosion. AGs were dissected, fixed and imaged by confocal microscopy. Glands were stained with DAPI (blue) to mark nuclei and an anti-Fas3 antibody (yellow) to mark cell boundaries. SCs express nuclear GFP, which is also present in the cytosol. Pairs of nuclei from binucleate SCs and MCs are indicated by green and red arrows respectively. **G.** Bar chart showing SC nuclear size relative to that of MC neighbours for glands expressing different transgenes in SCs under esgF/O^ts^ control, normalized to the ratio for controls. Note that all manipulations that decrease BMP signalling significantly reduce SC nuclear size. Genotypes for images are: *w; PBac[544*.*SVS-1]tkv*^*CPTI002487*^ (B); *w; esg-GAL4 tub-GAL80*^*ts*^ *UAS-FLP; UAS-GFP*_*nls*_ *actin>FRT>CD2>FRT>GAL4* combined with no other transgene (C); *P[TRiP*.*JF01485]attP2* (III) (D); *P[w*^*+*^ *UAS-Dad]* (II) (E); *P[TRiP*.*JF01263]attP2* (III) (F). ***P<0.001, Kruskal-Wallis test, n = 10. Scale bar for (B) is 10 μm, for all other images it is 20 μm.

Secondary cells (SCs) of the paired exocrine *Drosophila* male accessory glands (AGs) form multiple large intracellular secretory compartments [15,16] (Fig 1A). A minority of these compartments are Rab7-positive, acidic, mature late endosomal multivesicular bodies or lysosomes (MVBLs) [17]. The remaining compartments have been referred to as secretory vacuoles (SVs) [16]. Many, but not all, of these contain DCGs [16,17]. To date, the only characterised molecule identified within these DCGs is the homologue of angiotensin-I converting enzyme, ANCE [16].

Like their mammalian counterparts, the prostate and seminal vesicles, the AGs secrete a complex mixture of proteases, protease inhibitors and other glycoproteins into seminal fluid, which are transferred to females during mating [18]. The glands’ secretome modulates female behaviour, for example by increasing egg laying and feeding, and reducing the female’s receptivity to subsequent mating [19]. Many of these functions are attributed to sex peptide [20], which is secreted by the second epithelial cell type in the AG, the main cells (MCs).

More recent data also implicate SCs in modifying female behaviours. Abnormal development of SCs in males is associated with reduced ability to induce female egg laying and inhibit female receptivity [21,22]. MVBLs in SCs generate large numbers of intraluminal vesicles (ILVs) that are labelled by a GFP-tagged form of the human tetraspanin CD63, which is typically associated with secreted vesicles called exosomes in mammalian systems [17]. Indeed, CD63-GFP-positive vesicles are released into the AG lumen. Blocking exosome formation or trafficking regulators in adult SCs specifically affects the male’s ability to inhibit female remating [17].

Interestingly, post-mitotic adult SCs grow as males age, a process enhanced by mating [23]. Bone Morphogenetic Protein (BMP) signalling is essential for this growth, for formation and maturation of MVBLs and for exosome secretion [17]. It is also required for reprogramming of female remating behaviour, although it is unclear whether this is linked to its role in exosome secretion [23].

Here we show that Decapentaplegic (Dpp), the homologue of mammalian BMP ligands, BMP2 and BMP4, is packaged into DCGs of SCs and is the primary driver of BMP-dependent processes in SCs. We demonstrate that regulated DCG release involves the SNARE protein SNAP24 and is stimulated by mating. Secretion drives a rapid Dpp-dependent autocrine feedback mechanism and some paracrine signalling between SCs, which accelerates the replenishment of new DCG compartments in preparation for subsequent matings. Previously reported roles for BMPs in DCG secretion suggest that this autocrine mechanism may be shared by other higher eukaryotic cells, potentially offering a new route for modulating defective secretion in disease.

## Results

### Multiple BMP signalling components control SC growth

We previously showed that expression of a constitutively activated form of the Type I BMP receptor Thick veins, Tkv (Tkv^ACT^, containing a Q199D substitution) [24], drives SC growth and secretion, while knockdown of the co-Smad *Medea* (*Med)* [25], a BMP-dependent transcriptional regulator, or overexpression of the downstream BMP antagonist *Daughters against Decapentaplegic*, *Dad* [26], blocks adult SC growth and endolysosomal trafficking [23,17]. To determine which BMP receptors are expressed in SCs, we first analysed a yellow fluorescent protein- (YFP-) tagged *tkv* gene trap line, *tkv*^*CPTI002487*^ [27]. It was expressed in all SCs, primarily associating with the plasma membrane and small intracellular compartments distinct from the large SC compartments (Fig 1B). In addition, an antibody against the Type II BMP receptor Wishful Thinking (Wit) [28], which forms a heterodimer with Type I receptors, labelled a subset of the Tkv-YFP-positive intracellular compartments and the cell surface (Fig 1B). These data suggest that Tkv and Wit are normally expressed in SCs.

To test which BMP signalling genes normally control SC growth, they were knocked down with specific RNAis in adult SCs using the esgF/O^ts^ driver line [23]. In this line, a ubiquitously expressed, temperature-sensitive form of the GAL4 inhibitor GAL80 blocks *esg*-GAL4-dependent expression until the temperature is shifted to 28.5°C in newly eclosed virgin males, driving SC-specific RNAi and nuclear GFP expression in the AG.

We knocked down the Type I BMP receptors, *tkv* and *saxophone* (*sax*), and the Type II receptors, *punt* (*put*) and *wit*, in adult SCs, using two independent RNAis per gene, several of which have been used successfully in previous analyses of BMP signalling in other cell types (see Materials and Methods). SC growth was assessed in 6-day-old adults by measuring nuclear size relative to neighbouring MC nuclei. We have found previously that this provides a simple assay to gauge overall SC growth, because nuclear size typically correlates with SC size [23,17].

RNAi-induced silencing of each receptor, but not a control RNAi (targeting MC-specific *Sex Peptide*) significantly decreased relative SC:MC and absolute SC nuclear size compared to controls, demonstrating that they play non-redundant roles in regulating growth (Figs 1C, 1D and 1G, S1A–S1D and S2A–S2C). It was not feasible to gauge the level of knockdown in these experiments by RT-PCR of AG mRNA, because SCs represent about 2% of all the cells in the AG, and it is unclear whether the different BMP signalling components are exclusively expressed in SCs. However, Wit expression levels, as judged by immunostaining, were greatly reduced following SC-specific *wit* knockdown, indicating strong knockdown in this experiment (S2G and S2H Fig). Consistent with previous findings [23], downregulation of BMP signalling by knockdown of *Med* or *Mad*, the gene encoding Med’s binding partner, or by overexpressing Dad also significantly inhibited SC growth, while up-regulating BMP signalling through overexpression of Tkv^ACT^ increased growth (Figs 1E–1G, S1E, S1F and S2A–S2C). None of the UAS-linked constructs affected growth in the absence of a GAL4 driver (S2D Fig). We conclude that SCs express the signalling machinery required to transduce BMP signalling via more than one non-redundant receptor complex.

### BMP signalling regulates SV numbers, endolysosomal maturation and exosome secretion

**Fig 2:**
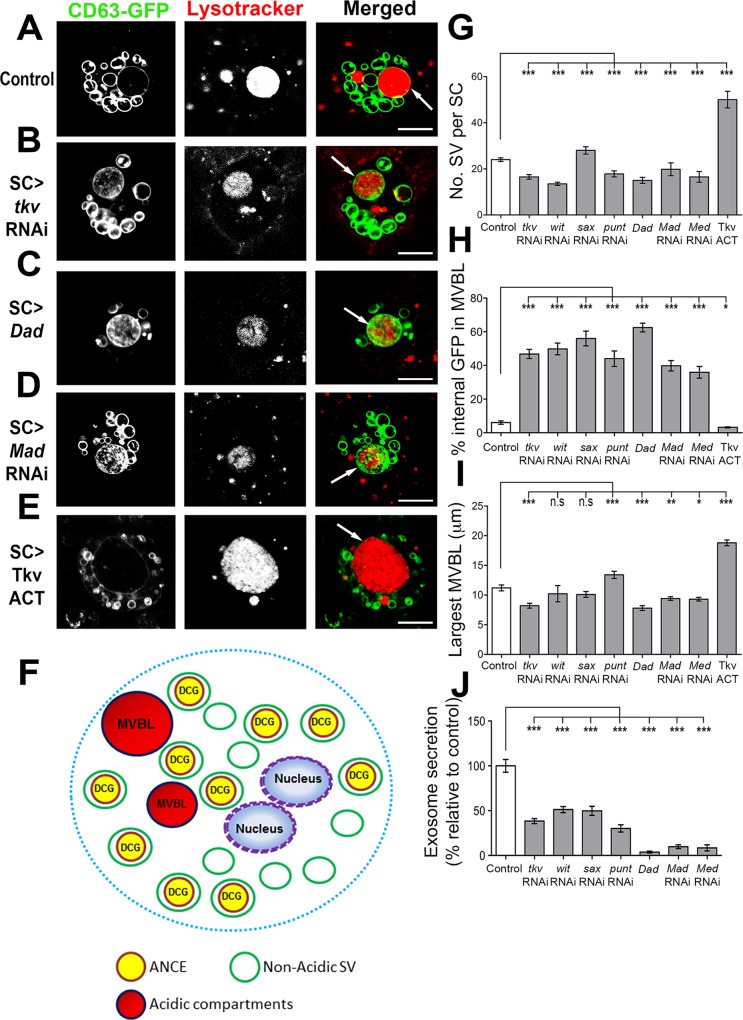
Multiple BMP signalling components in SCs regulate numbers of SVs, endolysosomal maturation, largest MVBL size and exosome secretion. **A-E.** SCs from 6-day-old males expressing CD63-GFP and either no additional transgene (A), or RNAis against *tkv* (B) or *Mad* (D), or overexpressing *Dad* (C) or an activated form of the *Tkv* receptor (Tkv^ACT^; E) throughout adulthood. They were stained with the acid-sensitive dye Lysotracker Red (red), and imaged under live conditions using confocal microscopy. Images represent a single z-plane, so do not show all SVs. **F**. Schematic representation of different intracellular SC compartments, showing large non-acidic compartments (SVs), most of which contain DCGs. **G**. The number of large CD63-GFP-positive SVs (>1μm diameter) per SC was counted. Inhibiting BMP signalling (except when using *sax*-RNAi) results in fewer SVs, while activating the pathway produces more. **H**. In control glands (A), GFP fluorescence is largely undetectable in the lumen of the largest, most mature MVBL (arrow). Inhibition of BMP signalling, however, results in significant accumulation of GFP fluorescent signal in the MVBL lumen. **I**. Reducing BMP signalling often reduces the diameter of the largest MVBL, while activating the pathway (by overexpressing Tkv^ACT^) increases the diameter. **J**. Inhibiting BMP signalling in SCs reduces the number of CD63-GFP-positive exosomes inside the AG lumen. Genotypes for images are: *w; UAS-CD63-GFP tub-GAL80*^*ts*^*; dsx-GAL4* combined with no other transgene (A); *P[TRiP*.*JF01485]attP2* (III) (B); *P[w*^*+*^ *UAS-Dad]* (II) (C); *P[TRiP*.*JF01263]attP2* (III) (D); *P[w*^*+*^ *UAS-Tkv*^*ACT*^*]* (E). The *w; UAS-CD63-GFP tub-GAL80*^*ts*^*; dsx-GAL4* line was employed to produce data in G-J. *P<0.05, **P<0.01, ***P<0.001, Kruskal-Wallis test, n = 10. Scale bar for all images is 10 μm.

We previously reported that blocking BMP signalling in SCs by overexpressing Dad decreases the total number of large non-acidic SVs, prevents MVBL maturation and inhibits exosome secretion [17]. We tested whether all BMP signalling components are similarly involved in these processes. A GFP-tagged form of the human exosome marker CD63 [29] labels all or most large (>1 μm diameter) internal SC compartments and exosomes released by SCs (Fig 2A and 2F) [17]. Since incorporating new GFP-tagged transgenes into the esgF/O^ts^ line was technically challenging, we employed the *dsx*-GAL4 driver, which is expressed only in SCs within the AG epithelium and has been used previously with UAS-CD63-GFP to mark SC exosomes [17]. Expressing Dad and Tkv^ACT^ with *dsx*-GAL4 produces the same effects as the esgF/O^ts^ driver on SC nuclear size (S2E Fig), suggesting similar levels of GAL4 expression with these two drivers. Furthermore, SC nuclear size was unaffected in 6-day-old males by expression of CD63-GFP under *dsx*-GAL4 control from eclosion onwards (S2F Fig), indicating that SC growth was not affected by expression of this marker.

RNAi knockdown of *tkv*, *put*, *wit*, *Mad*, or *Med* using *dsx*-GAL4 resulted in an overall decrease in SV number, while Tkv^ACT^ produced an increase (Figs 2B–2E, 2G and S3B–S3G). Surprisingly, both *sax*-RNAi lines appeared to increase SV numbers (Figs 2G, S3D and S3G), suggesting that this receptor might have a different effect to other signalling components on SC membrane trafficking.

Fluorescence of GFP fusion proteins, including CD63-GFP, is quenched by the acidic microenvironment of MVBLs [17,30]. Analysis of confocal z-sections through each SC’s largest MVBL in control 6-day-old flies showed that less than 10% of the total area was marked by ILV-associated fluorescent GFP in live glands (Fig 2A and 2H), even though anti-GFP antibody staining revealed that this compartment was full of cross-reacting GFP (S3A Fig). Silencing any BMP signalling component resulted in significantly increased accumulation of fluorescent CD63-GFP inside the largest MVBL (Figs 2B–2D, 2H, S3B–S3F and S3H). Conversely, Tkv^ACT^ expression significantly reduced the GFP signal (Fig 2E and 2H), supporting the idea that BMP signalling through multiple receptors promotes MVBL maturation.

In agreement with our previous findings [17], the size of the largest MVBL was also often reduced when BMP signalling was inhibited (Fig 2I and S3I Fig), consistent with a role in driving trafficking into this compartment. However, we found that knockdown of some BMP signalling components did not produce this phenotype (eg. both *punt* RNAis), perhaps because degradation and export of MVBL contents are reduced by these treatments as well as endolysosomal trafficking. Knockdown of all BMP receptors, *Mad* or *Med* in adult SCs resulted in a significant reduction in CD63-GFP-positive puncta in the AG lumen, which we have previously shown represent exosomes [17], as did overexpression of Dad (Fig 2J and S3J Fig). In conclusion, all *Drosophila* BMP receptors and major downstream transcriptional regulators are required to retain normal SV number, promote endolysosomal maturation and drive exosome release from SCs, demonstrating that this signalling pathway plays a key role in secretory trafficking events through multiple receptor combinations.

### BMP-dependent SC growth and membrane trafficking are primarily regulated via autocrine Dpp signalling

*Drosophila melanogaster* synthesises three BMP ligands, two of which are closely related to mammalian BMPs: Decapentaplegic (Dpp) is homologous to BMP2 and BMP4, and Glass bottom boat (Gbb) is more closely related to BMP5-8. The third ligand Screw (Scw) is a more distant BMP relative, which does not appear to be expressed in adults [31] and was therefore not analysed.

**Fig 3:**
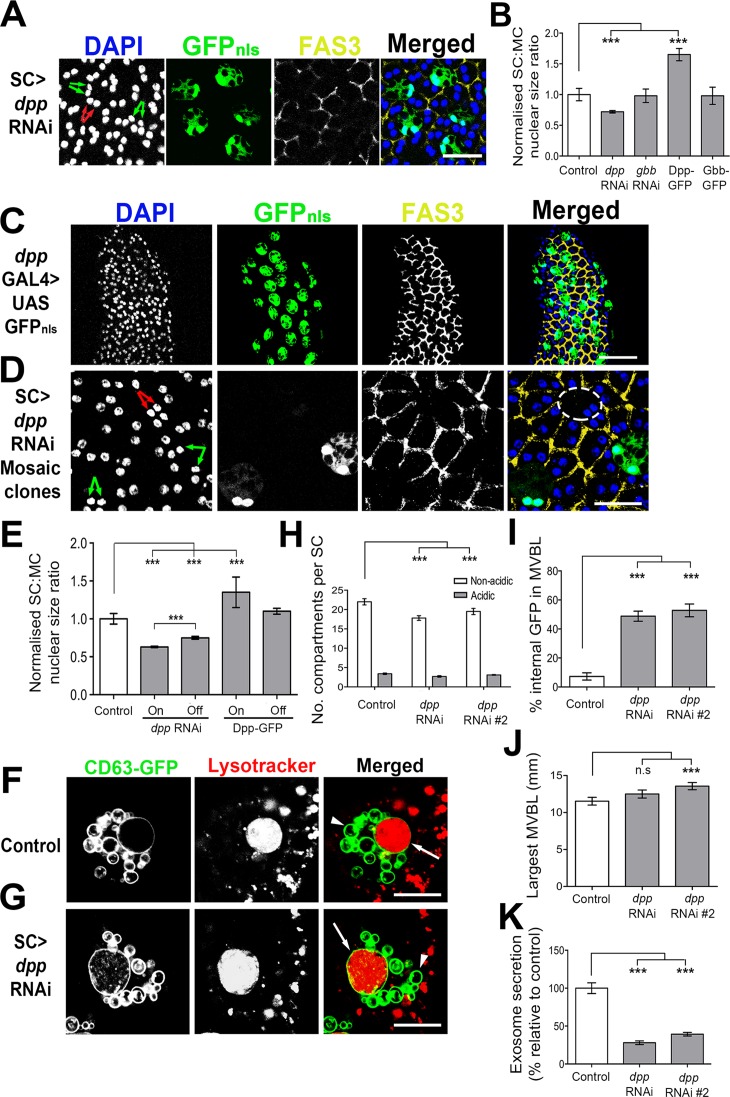
Autocrine Dpp regulates SC growth, SV number, endolysosomal trafficking and exosome secretion. **A**. Expression of *dpp*-RNAi during the first six days of adulthood using the esgF/O^ts^ driver reduces the size of SCs and their nuclei (green arrows) relative to MCs (red arrows). **B**. Relative SC:MC nuclear size for SCs expressing RNAis targeting *dpp* and *gbb*, or GFP-tagged Dpp and Gbb, revealing specific effects of Dpp on growth. **C**. *dpp*^*blk*^-GAL4 drives expression of a UAS-coupled nuclear GFP exclusively in SCs of the AG. **D, E.** Mosaic expression of *dpp*-RNAi or Dpp-GFP in a subset of SCs has a stronger effect on nuclear growth in expressing cells (on–green arrows) than in non-expressing (off–red arrows; white dashed circle) SCs, although *dpp* knockdown also reduces growth in the latter. **F-K.** Co-expression of *dpp*-RNAi with CD63-GFP using the *dsx*-GAL4 driver (G) reduces non-acidic SV number (eg., marked by arrowhead) and increases GFP fluorescence in largest MVBL (arrow; stained with Lysotracker Red) compared to controls (F); the statistical analysis of these changes for two independent RNAis is shown in H and I respectively. Knockdown of *dpp* either results in a small increase in the size of the largest MVBL or no significant size change (J), and reduces exosome secretion (K). Confocal images are from fixed glands (A, C, D) stained with DAPI (blue) and for Fas3 (yellow) or from living glands (F, G). Genotypes for images are: *w; esg-GAL4 tub-GAL80*^*ts*^ *UAS-FLP; UAS-GFP*_*nls*_ *actin>FRT>CD2>FRT>GAL4/P[TRiP*.*HMS00011]attP2* (A and mosaic in D; the esgF/O^ts^ driver was also used to generate data in E); *w; P[w*^*+*^ *UAS-GFP*_*nls*_*]; P[w*^*+*^ *dpp*^*blk*^*-GAL4]* (C); *w; UAS-CD63-GFP tub-GAL80*^*ts*^*; dsx-GAL4* combined with no other transgene (F) or *P[TRiP*.*HMS00011]attP2* (III) (G). ***P<0.001, Kruskal-Wallis test, n = 10. Scale bar for A, D is 20 μm, F, G, 10 μm, and for C, 50 μm.

Knockdown of either *dpp* or *gbb* in adult MCs using the MC-specific driver, *Acp26Aa*-GAL4 [32], suggested that neither gene controls SC growth in these cells (eg. S4F Fig). However, SC-specific silencing of *dpp*, but not *gbb*, resulted in a significant reduction in SC nuclear growth (Figs 3A and 3B, S4D, S4E, S4G and S4H), indicating that SC-expressed Dpp normally controls SC growth. Moreover, overexpression of GFP-tagged Dpp, Dpp-GFP [33], but not Gbb-GFP [34], in SCs increased SC growth (Figs 3B, S4A, S4D and S4E), even though both fusion proteins can activate BMP signalling in other tissues. Interestingly, overexpression of Dpp-GFP in MCs had no effect on growth of these cells, but did increase SC growth (S4C and S4F Fig).

To further investigate whether *dpp* is normally synthesised in SCs, we analysed the expression of a previously characterised *dpp*-GAL4 transgene, *dpp*^*blk*^-GAL4, which recapitulates endogenous *dpp* expression in several developmental scenarios [35,36]. In the adult AG, *dpp*^*blk*^-GAL4 drives UAS-GFP expression exclusively in SCs (Fig 3C). Although we cannot be certain that this reflects normal *dpp* expression, combined with the *dpp* knockdown data, we conclude that SCs are the primary source of SC-regulatory BMP activity in the AG.

To determine whether Dpp signals cell-autonomously or non-autonomously to control SC growth, *dpp*-RNAi-expressing SC mosaics were produced using the esgF/O^ts^ system [23]. Growth of those SCs expressing *dpp*-RNAi, which are GFP-positive, was most severely reduced (Fig 3D and 3E). However, SCs not expressing the RNAi were also affected, albeit to a lesser extent. Conversely, mosaic overexpression of UAS-*dpp-GFP* increased nuclear growth only in SCs expressing the transgene (Fig 3E and S4B Fig). We conclude that SC-expressed Dpp primarily regulates growth via an autocrine mechanism, but can also exert paracrine effects on other SCs.

To test whether Dpp, like other BMP signalling components, affects membrane trafficking events within SCs, it was knocked down in SCs expressing CD63-GFP during the first 6 days of adulthood. The number of SVs was reduced and endolysosomal maturation inhibited (Fig 3F–3I), mirroring effects seen with knockdown of other BMP signalling components. The size of the biggest acidic compartment, which is more variably affected by changes in BMP signalling (Fig 2I), was slightly enlarged with one *dpp*-RNAi treatment and unaffected by the other (Fig 3J). Dpp was required for the secretion of CD63-GFP-positive exosomes into the AG lumen (Fig 3K). Our results demonstrate that Dpp signals mainly in an autocrine way in SCs and is the primary regulator of BMP-dependent growth, SV number, endolysosomal maturation and exosome secretion in these cells.

### Dpp is stored in DCGs of SCs prior to secretion

**Fig 4:**
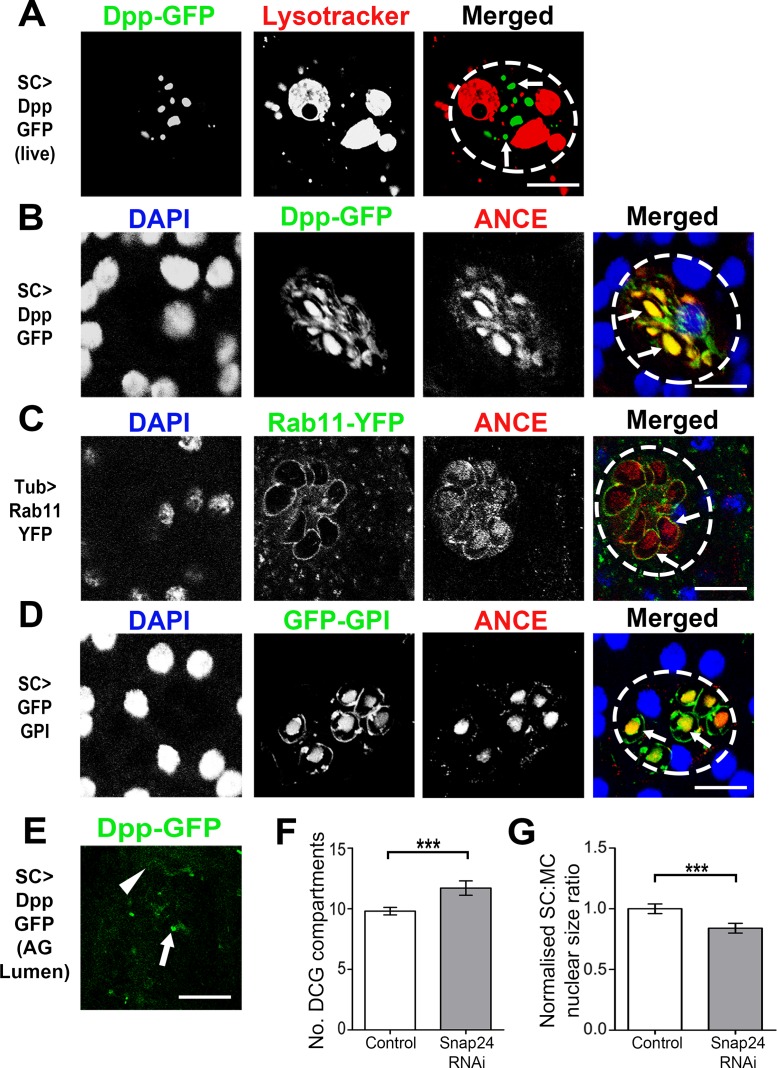
Dpp is stored in SC dense-core granules. **A**. Live image of Dpp-GFP-expressing SC stained with Lysotracker Red to identify acidic compartments. Note that Dpp-GFP localises to spherical structures (arrows) that are distinct from acidic compartments. **B**. Image of fixed SC, stained with anti-ANCE antibody (red) and DAPI (blue) after 24 h pulse of Dpp-GFP expression, reveals co-localisation of GFP fluorescence with ANCE-positive DCGs (arrows). **C**. Rab11-YFP-positive compartments in SCs from flies ubiquitously expressing this fusion protein under tubulin promoter control contain DCGs that stain positive for ANCE (red). **D**. Fixed SC expressing GFP-GPI and stained with anti-ANCE (red) and DAPI (blue), showing co-localisation of ANCE and GFP in DCGs (arrows). **E**. GFP-positive puncta (arrow) and filaments (arrowhead) are detected in the AG lumen when Dpp-GFP is expressed in SCs. **F, G.** SC-specific expression of *Snap24* RNAi in adults significantly increases number of GFP-GPI-labelled DCGs in 6-day-old virgin males (F; using *spi*-GAL4 driver), and reduces nuclear size (G; using esgF/O^ts^ driver). All images are from 3-day-old virgin males and individual SCs are outlined by dashed circles. Genotypes for images are: *w; tub-GAL80*^*ts*^*; dsx-GAL4/UAS-Dpp-GFP* (A, B, E); *w; tub-rab11-YFP* (C); *w; spi-GAL4 tub-GAL80*^*ts*^ *UAS-GFP-GPI/CyO* (D). ***P<0.001, Mann-Whitney *U* test, n = 10. Scale bar for A-D is 10 μm and E is 20 μm.

To determine how SC-expressed Dpp is trafficked and secreted, Dpp-GFP was overexpressed specifically in these cells. Live confocal imaging revealed that Dpp-GFP is concentrated in spherical structures resembling DCGs, but is typically absent from MVBLs (Fig 4A). Co-staining Dpp-GFP-expressing SCs with the DCG marker ANCE [16] confirmed that Dpp-GFP is mostly trafficked to DCGs (Fig 4B). Consistent with this, an RFP-tagged form of Dpp [37] primarily trafficked into CD63-GFP-positive SVs (S5A Fig). DCG localisation does not appear to be the result of overexpression of tagged Dpp, because even a short one hour pulse of Dpp-GFP expression is chased into DCG compartments over a subsequent12 h period (S5B Fig).

Previously, we reported that a subset of non-acidic SVs is marked by the slow recycling endosome marker Rab11 [17,38]. Co-staining SCs from flies expressing Rab11-YFP from a tubulin promoter [38] with an anti-ANCE antibody revealed that all ANCE-containing DCG compartments were lined with Rab11 (Fig 4C), indicating that DCGs are located in Rab11-positive compartments. Although we have previously reported that the tub-Rab11-YFP transgene suppresses formation of large MVBLs in SCs [17], the number of Rab11-YFP-positive compartments was identical to the number of ANCE-positive compartments in wild type cells from 3-day-old males (~10; S4I Fig). Therefore, the subset of SVs that contains DCGs appears to be unaffected by expression of this Rab fusion protein. Like ANCE, Dpp-GFP was secreted from SCs into the AG lumen (Fig 4E), from where it potentially exerts its paracrine effects on other SCs.

### DCGs can be marked by a glycophosphatidylinositol-anchored form of GFP

Since overexpressing Dpp-GFP in SCs over extended periods has a significant effect on membrane trafficking and cell growth (Fig 3E), we investigated alternative markers to label DCG compartments in living cells. We found that when expressed in SCs, UAS-regulated GFP-GPI [39], a form of GFP that binds to the outer leaflet of lipid bilayers via a glycophosphatidylinositol (GPI) anchor, also traffics to the limiting membrane of DCG compartments and surprisingly concentrates in ANCE-positive DCGs, even though they do not appear to contain membranes (Fig 4D).

Since we found it difficult to make a line containing both UAS-*GFP-GPI* and *dsx-*GAL4, we combined the GFP-GPI transgene with a ubiquitously expressed GAL80^ts^ construct and *spitz*-GAL4 (*spi*-GAL4), which drives GAL4 expression strongly and selectively in SCs of the AG (Fig 4D). *spi*-GAL4 induces similar nuclear growth phenotypes to *dsx*-GAL4 and esgF/O^ts^ when combined with UAS-*Dad* and UAS-*Tkv*^*ACT*^ constructs (S2E Fig). It also has a similar effect to *dsx*-GAL4 on largest MVBL size when combined with these regulators of BMP signalling (S3K Fig). These data indicate that *spi*-GAL4 promotes UAS-dependent gene expression at roughly the same level as the other two drivers. In virgin 3-day-old males, GFP-GPI expressed under *spi*-GAL4 control from eclosion onwards marked the same number of DCGs as ANCE and Rab11-YFP (S4I Fig). Furthermore, GFP-GPI overexpression with *spi*-GAL4 did not significantly affect SC nuclear growth (S2F Fig) or MVBL numbers (S6A Fig) over the first six days of adulthood compared to other genotypes. However, it did increase the size of the largest MVBL when compared to SCs from *w*^*1118*^ control males, albeit not as much as CD63-GFP overexpression (S6B Fig). In fact, we also found that largest MVBL size was increased in SCs from esgF/O^ts^ males compared to *w*^*1118*^ control males (S6B Fig), suggesting that this specific cellular property may be modulated by overexpression of any GFP-marked transgene. However, aside from this change in MVBL size, we conclude that the *spi*-GAL4 UAS-*GFP-GPI* reporter line has no obvious effect on normal SC biology.

In undertaking this analysis, we noticed that the total number of SVs in CD63-GFP-expressing SCs was considerably greater than the number of DCG compartments in GFP-GPI-expressing cells (24 ± 0.8 versus 9.8 ± 0.3), and that the former compartments appeared smaller than the latter. To determine whether CD63-GFP overexpression altered DCG compartments in SCs, we co-expressed this transgene with GFP-GPI. This combination increased the number of GFP-GPI-positive DCGs in SCs from 6-day-old males (S6C–S6E Fig), suggesting that although compartment identity is not obviously defective in CD63-GFP-overexpressing SCs, the number of large non-acidic compartments is increased (and their size reduced). We speculate that this reflects an increased rate of trafficking through the secretory and endolysosomal systems. By contrast, when expressed alone, the GFP-GPI transgene does not appear to affect the size or number of large compartments in SCs.

Since BMP signalling stimulates SC growth even in virgin males, we reasoned that Dpp-containing DCGs must be steadily released from SCs during adulthood. SNARE proteins have been implicated in regulating fusion of DCG compartments and synaptic vesicles to the plasma membrane in several eukaryotic systems [9,40]. In the *Drosophila* salivary gland, the granule-associated SNARE, SNAP24, a homologue of mammalian SNAP25, is involved in glue granule release [41,42]. Expression of an RNAi targeting *Snap24* in adult SCs significantly increased the number of GFP-GPI-positive DCGs, suggesting that SNARE-mediated fusion events are required for normal DCG release in virgin males (Fig 4F). Nuclear growth was also decreased in these cells (Fig 4G), as would be expected when BMP signalling is reduced.

### Activation of BMP signalling in SCs is detected by an anti-pMad antibody

**Fig 5:**
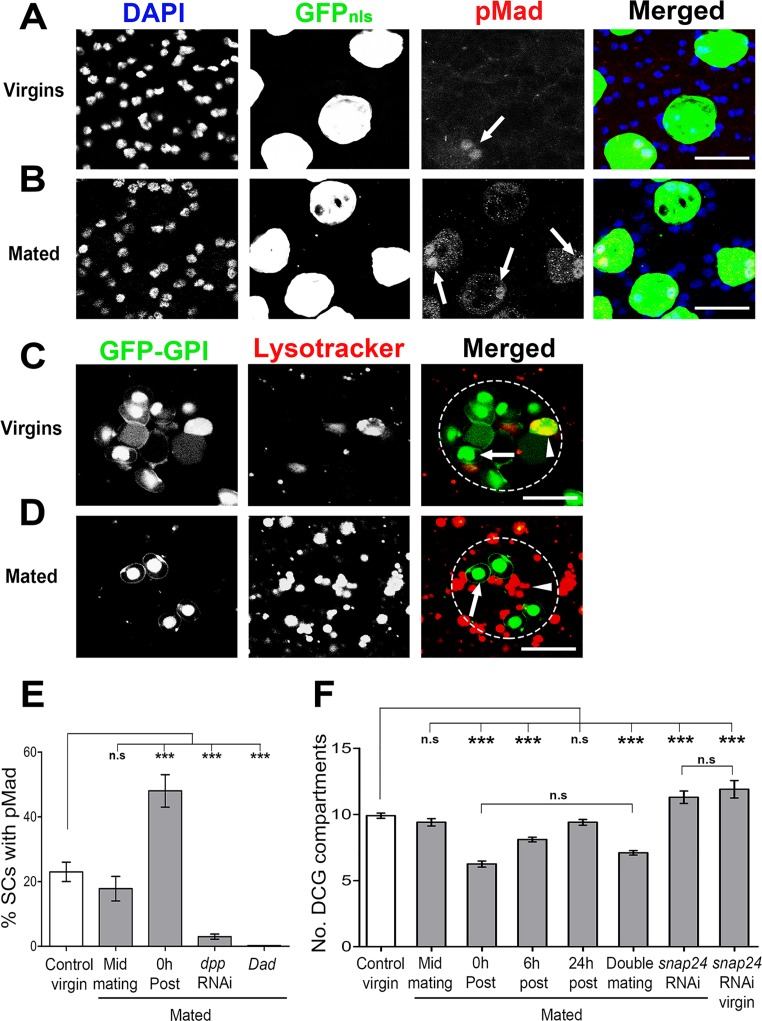
DCGs are released from SCs during mating, activating BMP signalling. **A, B**. SCs from 6-day-old esgF/O^ts^ virgin males (A) and from males immediately after mating (B) were fixed and stained with an anti-pMad antibody (red) and DAPI (blue), revealing that the proportion of SCs with detectable nuclear pMad is higher in mated animals. **C, D.** Immediately after mating, living SCs (D) have less GFP-GPI-labelled DCGs than virgins (C). Image shows a single z-plane of gland stained with Lysotracker Red; not all compartments are in the focal plane. Note that the largest MVBL in (C; arrowhead) contains GFP, probably because of fusion between a DCG compartment and the MVBL [17]. **E**. Graph shows proportion of SCs with nuclear pMad in 6-day-old virgin, and mated males (dissected 8 min into mating [Mid] and immediately after mating), and mated males expressing *dpp*-RNAi and Dad in SCs from eclosion onwards using the *w; esg-GAL4 tub-GAL80*^*ts*^ *UAS-FLP; UAS-GFP*_*nls*_ *actin>FRT>CD2>FRT>GAL4* driver. **F**. Graph shows number of GFP-GPI-positive DCG compartments in 6-day-old virgin and mated males (using the *w; spi-GAL4 tub-GAL80*^*ts*^ *UAS-GFP-GPI* driver line; Double is twice mated in 2 h), and at different times after single mating in control SCs. Compartments were also counted in SCs expressing *Snap24* RNAi post-eclosion in virgins and immediately after mating. Labelled compartments were counted using a complete z-series for each cell. Genotypes for images are: *w; esg-GAL4 tub-GAL80*^*ts*^ *UAS-FLP/+; UAS-GFP*_*nls*_ *actin>FRT>CD2>FRT>GAL4/+* (A, B); *w; spi-GAL4 tub-GAL80*^*ts*^ *UAS-GFP-GPI/CyO* (C, D).***P<0.001, Kruskal-Wallis test, n>15. Scale bar for A-B is 20 μm and C-D is 10 μm.

Activation of BMP signalling leads to phosphorylation of cytosolic Smad proteins, which then translocate to the nucleus to regulate gene transcription [43]. Nuclear phospho-specific anti-Mad antibody staining is a commonly used readout for BMP pathway activity in flies [44,45]. Although no pMad signal was observed in MCs, nuclear pMad was detected in some SCs from virgin 3- and 6-day-old esgF/O^ts^ males (9% and 20% respectively) (Figs 5A, 5E and S7D). Knockdown of BMP signalling components in SCs strongly reduced the number of pMad-positive nuclei (S8A Fig), suggesting BMP signalling is sporadically activated in these cells, perhaps upon DCG release.

To further validate pMad staining as a marker for BMP signalling in SCs, we overexpressed either Dpp-GFP or Tkv^ACT^ in SCs. This induced detectable nuclear pMad staining in all SCs and also in those SCs expressing Dpp-GFP in clones, while no nuclear pMad staining was observed in SCs when BMP signalling was blocked by overexpressing Dad (S4A, S4B and S7A–S7C Figs). MC-specific expression of Dpp-GFP had no effect on MCs themselves, but increased pMad levels in SCs (S4C Fig). Therefore pMad staining marks BMP signalling activity in AGs. Although all SCs can respond to BMPs when Dpp is overexpressed, there is only stochastic activation of signalling in SCs in normal virgin males.

### Mating induces rapid DCG compartment release from SCs resulting in activation of BMP signalling

When males mate, part of the luminal content of the AG is transferred to females. Western blot analysis of reproductive tracts from females mated with males expressing GFP-GPI in SCs revealed that this marker is transferred during mating (S7E Fig), consistent with previous findings using the DCG marker ANCE [17]. We hypothesised that DCG secretion from SCs might increase to restore luminal content in preparation for subsequent matings.

Interestingly, when GFP-GPI-expressing SCs of 6-day-old males were imaged immediately after a single mating, approximately four GFP-positive DCG compartments had been lost (Figs 5C, 5D and 5F), indicating that DCGs are secreted upon mating. Approximately two of these compartments were replenished within 6 h and by 24 h, the original number of DCGs was restored (Fig 5F). When flies expressing *Snap24*-RNAi in SCs were mated, no decrease in DCG numbers was observed compared to virgins, indicating that *Snap24* is required for mating-induced DCG release (Fig 5F). AG luminal contents are only transferred to females during the second half of the ~20 min mating process [46,47]. Males separated after only 8 minutes of mating did not show a significant reduction in GFP-GPI-positive DCGs (Fig 5F), suggesting that secretion coincides with transfer of AG luminal content.

Since mating stimulates increased BMP-dependent growth of SCs [23], we reasoned that this might result from release of Dpp-containing DCGs during mating. To test this hypothesis, control 3- and 6-day-old esgF/O^ts^ males were mated and their AGs immediately dissected and stained for pMad. Although we observed no significant change in nuclear pMad staining between virgins and males separated 8 min into mating, there was a significant increase in the number of pMad-positive SCs immediately after mating in both 3- (S7D Fig) and 6-day-old (Fig 5E) males with approximately 50% of all SCs stained compared to 10% and 20% respectively in virgins. This effect was completely suppressed by expressing *dpp* RNAi or Dad in SCs (Fig 5E). These results indicate that Dpp contained within DCGs is released upon mating and stimulates BMP signalling via a mechanism that requires *Snap24*, and that ultimately promotes increased SC growth.

### Autocrine Dpp signalling is required to replenish DCGs after mating

**Fig 6:**
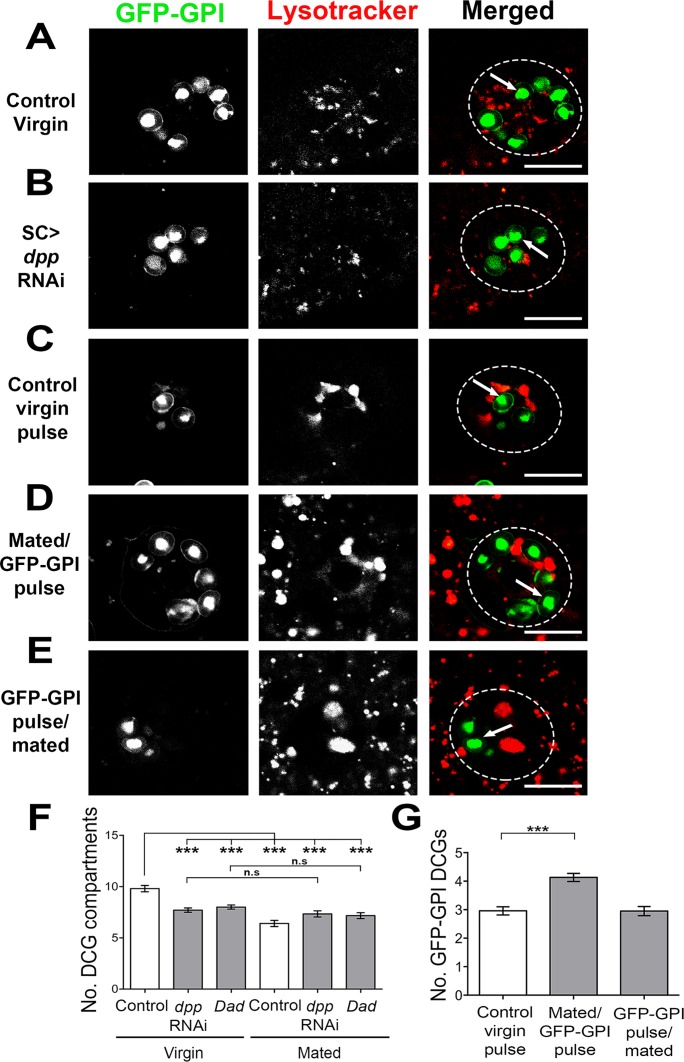
Mating rapidly accelerates the rate of new DCG compartment formation. **A, B**. Single z-plane images of SCs from 6-day-old virgin males labelled with GFP-GPI, either co-expressing (B) or not expressing (A) *dpp*-RNAi. Reducing Dpp signalling decreases the number of labelled DCGs (arrow). **C-E**. 6-day-old males were shifted to 28.5°C to induce GFP-GPI expression for 16 hrs in virgins (C) or immediately after (D) or before (E) mating. Their AGs were dissected and imaged; each image is from a single z-plane. **F**. Graph showing number of GFP-GPI-positive DCGs in SCs from either 6-day-old virgin or mated control flies or flies in which BMP signalling is inhibited. There is no decrease in total DCG number in SCs expressing either *dpp*-RNAi or *Dad* after mating. **G**. Graph showing GFP-positive DCG number after a 16 h pulse of GFP-GPI in SCs from 6-day-old males, as in C-E above. Genotypes for images are: *w; spi-GAL4 tub-GAL80*^*ts*^ *UAS-GFP-GPI* combined with no other transgene (A, and pulse-labelled in C, D, E) or *P[TRiP*.*HMS00011]attP2* (III) (B). The *w; spi-GAL4 tub-GAL80*^*ts*^ *UAS-GFP-GPI* line was used to generate data in F and G. ***P<0.001, Kruskal-Wallis test, n = 15. Scale bar for A-E, 10 μm.

SC-specific BMP signalling, which is required to fully inhibit female receptivity after mating [17], is also needed to maintain normal numbers of CD63-GFP-marked SVs, some of which contain DCGs. We tested more directly whether BMP signalling controls the number of DCGs in virgin flies using the GFP-GPI marker, which unlike CD63-GFP, does not noticeably affect DCG numbers in SCs when overexpressed (S4I and S6E Fig). Inhibiting the BMP pathway by knockdown of *dpp*, BMP receptors, *Mad* or *Med*, or by overexpression of *Dad* in adult SCs reduced the number of DCGs by about 20% (Figs 6A, 6B, 6F and S8B), except in the case of *sax*, which also has a unique effect on SV number in CD63-GFP-overexpressing SCs (Fig 2G and S3G Fig). These data indicate that normal biogenesis of DCGs is dependent on BMP signalling. To test whether mating-dependent DCG secretion also requires BMP signalling, we analysed flies after a single mating. There was no significant decrease in DCGs between mated and virgin flies when BMP signalling was inhibited by *dpp* knockdown or Dad overexpression (Fig 6F), suggesting that DCG release is blocked, perhaps because the compartments have failed to mature normally.

One explanation for the rapid replenishment of DCGs after mating is that BMP signalling upregulates biosynthesis of new DCG compartments. To test this, we conducted a series of experiments using pulses of GFP-GPI expression. A 16 h pulse of GFP-GPI labelled 3 ± 0.1 DCG compartments in virgin males (Fig 6C and 6G). When the 16 h GFP-GPI pulse occurred immediately after mating, there was a significant increase in the number of labelled DCGs (4 ± 0.1; Fig 6D and 6G), supporting a model whereby mating accelerates cargo loading into new DCGs. An alternative explanation is that some DCG compartments formed during the 16 h pulse are secreted in virgins, but not after mating. In mammals, immature DCGs are typically not secreted, but must undergo a stepwise maturation process involving luminal acidification by the v-ATPase and selective removal of non-DCG proteins prior to exocytosis [1,4]. To test whether pulse-labelled DCG compartments could be secreted from SCs upon mating, we induced GFP-GPI for 16 h before mating, then dissected the AGs immediately after mating. We did not observe any decrease in the number of labelled DCG compartments compared to virgin males (Fig 6E and 6G), indicating that GFP-GPI is trafficked over a 16 h period to new immature compartments, which cannot be immediately secreted. Indeed, if males expressing GFP-GPI throughout adulthood were remated within two hours for a second time, no additional DCGs were released (Fig 5F), suggesting that normally only about 4 DCGs are sufficiently mature to be secreted.

**Fig 7:**
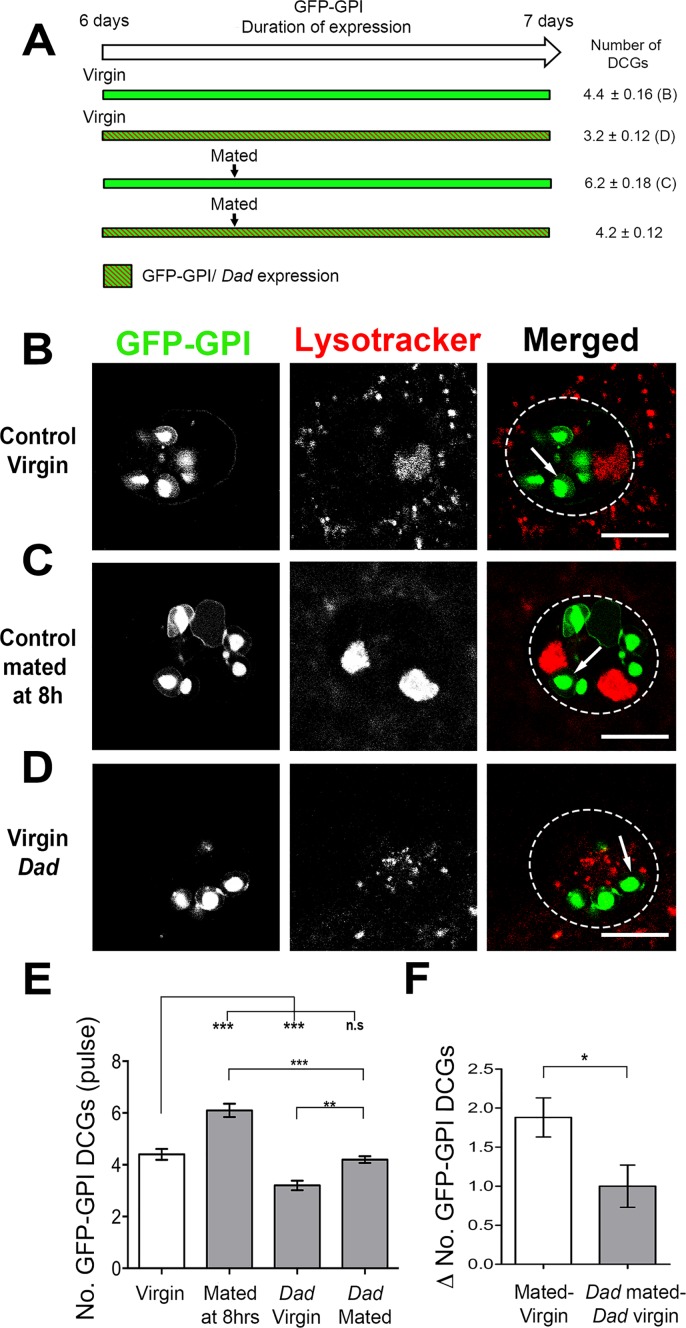
Rapid replenishment of DCGs after mating is BMP-dependent. **A**. Schematic representation of pulse-chase experiments shown in B-F, indicating the duration of GFP-GPI and *Dad* overexpression and timings of mating events. **B-D**. 6-day-old flies were shifted to 28.5°C for 24 h to allow expression of GFP-GPI in virgins (B) or in males mated 8 h after the start of the pulse (C). The number of GFP-GPI-labelled DCGs in SCs was reduced in virgin males co-expressing Dad (D). **E.** Graph shows a significant increase in the number of labelled DCGs if males are mated at 8h during a 24 h GFP-GPI pulse. The number of DCGs labelled in virgin and mated males is reduced if *Dad* is co-expressed. **F**. The increase in labelled compartments after mating is also reduced by Dad co-expression. The *w; spi-GAL4 tub-GAL80*^*ts*^ *UAS-GFP-GPI* line was used to generate data in E and F. *P<0.05, ***P<0.001, Kruskal-Wallis test, n = 15. Scale bar in B-D, 10 μm.

To assess whether the acceleration of DCG biosynthesis after mating involved BMP signalling, we co-overexpressed Dad during a 24 h GFP-GPI pulse to test whether this might induce sufficient inhibition of signalling to affect new DCG formation. Mating control flies 8 h after starting a 24 h GFP-GPI pulse increased DCG numbers when compared to virgins expressing GFP-GPI for the same duration (4.4 ± 0.2 DCGs in virgins versus 6.2 ± 0.2 DCGs in mated flies; Fig 7A–7C and 7E). However, when Dad was co-expressed with GFP-GPI in virgin flies for 24 h, the number of labelled DCGs was significantly reduced (Fig 7D and 7E), suggesting that BMP signalling is required for biogenesis of new immature DCGs. When we overexpressed GFP-GPI with Dad for 8 h, then mated male flies once and continued the pulse for a further 16 h (24 h in total) (Fig 7A), we observed a marked decrease in the number of labelled DCGs in comparison to mated control flies (Fig 7E). The number of marked DCGs was slightly higher than in Dad-expressing virgins, presumably because BMP signalling is not fully inhibited by a 24 h pulse of Dad. However, the difference between mated and virgin DCG numbers in Dad-expressing versus control males was significantly reduced (Fig 7F), demonstrating that BMP signalling is required to drive increased biosynthesis of new DCGs after mating.

## Discussion

**Fig 8:**
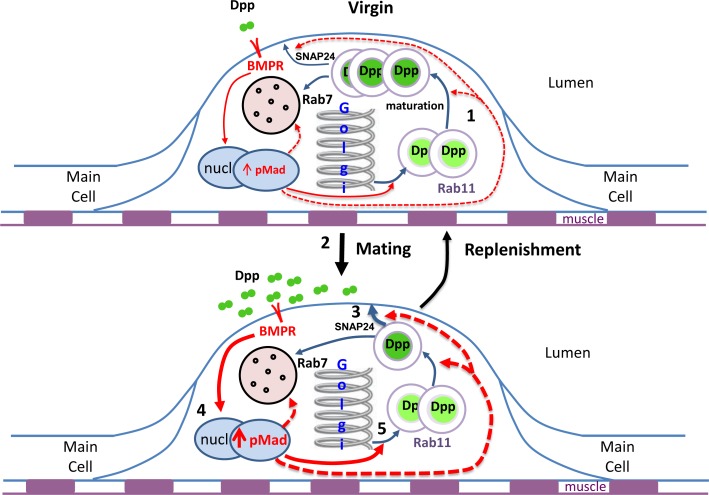
Model to explain autocrine regulation of DCG replenishment by BMP signalling in SCs. Schematics of a single SC immediately before and after mating. (1) In virgin males, Dpp is trafficked to and stored in DCGs. Sporadic release of these DCGs activates BMP signalling, and sustains a basal level of growth, DCG biogenesis and exosome secretion. (2) During mating, about 4 mature DCGs are released (3), resulting in an increase in BMP signalling (4), primarily via an autocrine mechanism and probably in pulses. This stimulates growth, but also increases biosynthesis of new DCG compartments (5; solid arrow), ensuring that the total number of DCGs is fully replenished within 24 h. Dashed arrows highlight other parts of the secretory/endolysosomal system that might be affected by altered BMP signalling. Previous data (Corrigan et al., 2014) and data presented here suggest that long-term elevated BMP signalling enhances endolysosomal trafficking.

Dense-core granule secretion is a fundamental and evolutionarily conserved process by which glandular epithelia release their bioactive contents in a regulated fashion. Importantly, to be sustained under different physiological conditions, this regulated output must be matched to new biogenesis. Here we show that *Drosophila* SCs with their large and easily counted DCGs provide a powerful new *in vivo* system to study this process. We demonstrate a critical role for BMP signalling in co-ordinating biogenesis and release, primarily via an autocrine mechanism. This signalling upregulates formation of new DCGs after rapid mating-induced secretion (Fig 8), a process that may be evolutionarily conserved.

### DCG release from SCs is accelerated during mating

We find that adult SCs from virgin males consistently contain approximately 10 Rab11-positive DCG compartments, as well as a number of other non-acidic compartments that do not contain DCGs and whose Rab signature is yet to be established. These other compartments are most clearly visualised using the CD63-GFP marker, which also labels DCG compartments and MVBLs. However, our study suggests that after expression in adult SCs for 6 days, this marker increases the number of SVs and DCG compartments, while reducing their size and increasing MVBL size. Therefore we have not yet been able to accurately determine the normal total number of SVs in SCs. Although these findings suggest that some aspects of secretion, including exosome secretion, may be modulated by CD63-GFP activity, it is important to emphasise that vesicles are observed inside wild type SC compartments by transmission EM [17], and are therefore not merely induced by CD63-GFP marker expression.

Our data further suggest that DCG compartments are turned over continuously. Expression of GFP-GPI in pulses indicates that one new immature DCG is formed about every 6 h. Furthermore, BMP signalling is sporadically activated in virgin males in a process requiring Dpp expression in SCs (Fig 5). Pancreatic β-cells, parotid and other glandular cells also have a basal constitutive-like secretory pathway [48,49].

Mating lasts about 20 min in flies. We find that approximately four DCG compartments are lost from each SC during the second half of this period (Fig 5). Newly-formed DCGs that are labelled during a 16 h GFP-GPI pulse are not released during mating, suggesting that only mature compartments are secreted in a regulated fashion, mirroring mammalian DCG secretion [1]. Indeed, since additional DCGs are not released after a second mating (Fig 5F), only about four DCGs normally appear to be sufficiently mature to be released. Knockdown experiments revealed that both constitutive and regulated secretion of DCGs is dependent on the SNARE protein, SNAP24 (Figs 4 and 5F), which has been shown to control regulated secretion from the fly salivary gland [41]. This function appears to be very broadly conserved in evolution, since the mammalian SNAP24 homologue, SNAP-25, is also implicated in insulin secretion from rat pancreatic β-cells [50] and DCG release from other secretory cells [9].

Other observations also suggest further parallels between SCs and other secretory cells. For example, DCGs contain proteases involved in processing bioactive secretory products; in SCs, ANCE has been proposed to play a role in this process [16]. Although multiple Rabs are implicated in DCG formation and secretion, homologues of Rab11, a key signature of DCGs in SCs (Fig 4C), have been highlighted in the secretory control of several mammalian DCGs, including pancreatic acinar cell zymogen [51] and β-cell insulin [52] granules. Finally, cholesterol-rich membranes and lipid raft structures, which associate with GPI-anchored proteins like GFP-GPI, are involved in mammalian DCG formation [7].

Ultimately GFP fluorescence associated with GFP-GPI in SCs traffics into the DCG core, even though there do not appear to be membranes inside these structures [16]. This could be explained in a number of ways. For example, the GPI anchor may be removed in DCG compartments to permit release into the dense core; indeed, mammalian ANCE (or ACE) has been reported to exhibit GPIase activity [53], although this finding has subsequently been challenged [54]. Alternatively, the GPI fusion protein may lose its association with the limiting membrane of the compartment membrane or much of the overexpressed GFP may lack a GPI anchor because it saturates the GPI anchor synthesis enzymes. It will therefore be interesting to test whether other GPI-anchored proteins expressed at more physiological levels also behave in this way in this and other systems. However, overall, the similarities in regulation of SC and mammalian DCGs indicate that SCs provide a powerful new *in vivo* model to genetically dissect fundamental aspects of DCG biogenesis.

### BMP signalling is increased during mating

Our findings that *dpp*^*blk*^-GAL4 drives gene expression only in SCs of the AG and that SC-specific knockdown of *dpp* has a strong effect on BMP-mediated SC functions (Fig 3), clearly show that Dpp expression from SCs plays a central role in controlling the biology of these cells. Staining with an anti-pMad antibody reveals the sporadic activation of BMP signalling in SCs, and an increase in cells with activated signalling during mating. Although it seems that all SCs release DCGs during mating and that overexpressing Dpp-GFP in SCs can activate BMP signalling in all expressing cells (S4A Fig), only about 50% of SCs from mated males contain nuclear pMad at any specific time, suggesting that activation after DCG release may be short-lived.

Interestingly, we previously found that a *Dad-GFP* transgene, which has been used as a BMP signalling reporter in other *Drosophila* cell types [55], is active in all SCs and at lower levels in MCs [23]. Its expression was not obviously increased by mating. It is unclear whether this reporter activity in SCs reflects cumulative activation of BMP signalling in SCs and/or non-BMP-dependent expression. However, since normal SC growth is primarily regulated cell-autonomously by BMP signalling [23,17] (Fig 3), we conclude that autocrine BMP signalling is probably independently activated in a pulsatile way in every SC as adults age. Phenotypic analysis of SC-specific *dpp*-RNAi expression in mosaics suggests that Dpp can also have paracrine effects on other SCs, but these cannot compensate for the effects of cell-autonomous loss of BMP signalling. Surprisingly, mosaic expression of Dpp-GFP does not affect growth of non-expressing SCs. One possible explanation is that this fusion protein is not released from SCs or detected by neighbouring SCs in the same way as wild type Dpp, although it does function normally in other cellular contexts [33]. Alternatively, there may be a threshold requirement for paracrine Dpp signalling or negative feedback signalling between SCs, while as more autocrine Dpp is produced, BMP signalling continues to increase.

Given the autocrine and secretion-dependent nature of BMP signalling in SCs, it is critical that Dpp is separated from its receptors prior to release. We found that at least one Type I and one Type II receptor (Tkv and Wit respectively) are trafficked to compartments that are distinct from the Rab11-positive DCGs. Unfortunately, we could not use a previously characterised fluorescence-based reporter for Tkv activation, TIPF [37], to investigate receptor activation further, because its expression under either UAS or ubiquitin promoter control activated BMP signalling constitutively and increased SC growth. Interestingly, work in *C*. *elegans* has shown that Type I and Type II receptors are separated after endocytosis [56], potentially terminating signalling. Our analysis suggests that Tkv and Wit may be in the same intracellular compartments in SCs, but their separation from Dpp presumably prevents continued activation.

One key question that now needs to be addressed is how DCG secretion is stimulated upon mating. One possibility is that during mating, the physical forces induced by peristaltic movement of the muscular sheath surrounding the AG could stimulate the fusion of DCG compartments docked close to the SC plasma membrane. Alternatively, a neuropeptide circuit has recently been identified in males that is critical for sperm and seminal fluid transfer and innervates cells in the AG [57]. It will be important to test whether this circuit makes connections with SCs or can stimulate DCG secretion through diffusion of the neurotransmitter(s) it releases.

### BMP signalling and the regulation of DCG biology in SCs

Overall our data support a model in which DCG-loaded Dpp acts primarily as an autocrine signal that matches regulated release of DCG compartments to new biogenesis (Fig 8). New DCG formation occurs continuously, even in virgin males, to replenish the constitutive release of these compartments. Upon mating, the rate of biogenesis is increased in a BMP-dependent fashion, so that two of the four DCGs lost at mating are replaced within 6 h. Blocking BMP signalling in SCs reduces biogenesis of new DCG compartments in both virgin and mated males, but also inhibits release of DCGs, which may explain why the number of DCGs in knockdown cells are only reduced by about 20% (Fig 6F and S8B Fig). One possible interpretation is that BMP signalling also controls DCG compartment fusion to the plasma membrane. However, currently we favour a model in which BMP signalling regulates maturation of DCGs, so they can be released upon stimulation. Indeed, it appears that only some DCGs are sufficiently mature to be secreted in normal SCs, because rapidly mating males a second time does not lead to further DCG release (Fig 5F). Identification of markers for DCG maturation status will help to test this specific aspect of our model further.

How could BMP signalling be driving elevated rates of DCG biogenesis? Downstream Smad signalling primarily regulates gene transcription. Previous studies in pancreatic beta-cells have revealed that BMP signalling induces insulin secretion and increases levels of many transcripts associated with the secretory process, such as the *Snap24* homologue, *SNAP25* [14]. Pheochromocytoma cells also express higher levels of the DCG assembly protein Chromogranin A in response to secretion [11]. Since SCs constitute only a small proportion of cells in the AG, we have not been able to use qRT-PCR methods to determine whether secretory regulators like *Snap24* are induced by mating or BMP signalling activation in SCs. We will need to identify SC-specific BMP target genes to study this in the future.

### BMP signalling co-ordinately controls compartment biogenesis, endolysosomal trafficking, exosome secretion and growth in SCs

Interestingly, we have found that BMP signalling has a role in several biosynthetic processes in SCs. We have employed three different GAL4 drivers to analyse BMP functions in SCs, esgF/O^ts^, *dsx*-GAL4 and *spi*-GAL4, which produce similar BMP-mediated effects on SC nuclear growth, and secretory and endolysosomal trafficking. Although expressed selectively in SCs within the AG, none of the drivers we use are only expressed in SCs within adult flies. Importantly, the consistent phenotypes induced with different drivers (eg. S2E Fig) therefore supports our conclusion that effects are induced by cell-autonomous changes in BMP signalling and not by altered signalling in other tissues.

Signalling appears to involve multiple BMP receptors working in a non-redundant way ([17] and this study) and acting through Mad activation, since knockdown of each receptor reduces numbers of pMad-positive SCs by about 75% (S8A Fig). Both Type I [58] and Type II [59] receptors have previously been shown to act non-redundantly in other *Drosophila* cell types. In SCs, knockdown of different BMP receptors can produce different effects; most notably *sax* RNAi did not reduce SV or DCG number in the same way as inhibiting other BMP signalling components (Figs 2G, S3G and S8B). Sax has previously been reported to have both positive and negative effects on BMP signalling through its different responses to Dpp and Gbb ligands [58]. To date, our knockdown analyses have not revealed a function for Gbb in SCs, although it appears to be expressed strongly in the AG [31]. Additional tests are required to determine whether Gbb might play a role in specific aspects of SC membrane trafficking.

In addition to controlling secretory and endolysosomal trafficking, BMP signalling also regulates SC nuclear size. Although in some *Drosophila* post-mitotic cells, like larval salivary gland cells and ovarian follicle cells, nuclear growth is achieved through endoreplication of the genome, nuclei can grow in other organs, such as the eye, without replicating the genome [60]. We previously were unable to detect new DNA synthesis in adult SCs using bromodeoxyuridine (BrdU) labelling [23], although we cannot exclude the possibility that this merely reflects the relatively low sensitivity of this method.

Another important consideration is whether the different effects of BMP signalling on SCs are mechanistically linked. For example, could the changes in MVBL maturation and exosome secretion observed when BMP signalling is inhibited result from BMP-dependent modulation of DCG membrane trafficking, or vice versa? The compartment phenotypes induced by blocking BMP signalling are consistent with a general reduction in membrane flux through the secretory and endosomal systems in SCs. However, a better understanding of the DCG, MVBL and exosome biogenesis pathways in these cells will be required to determine whether these processes are interdependent in other ways. For example, it will be important to determine how vesicles formed inside endosomal compartments are released into the AG lumen as exosomes, and whether the acidification of maturing DCG compartments reported in other systems [1,4] takes place in SCs and involves cross-talk with more acidic late endosomal compartments. Interestingly, cells within glands and neurons involved in regulated secretion are also reported to communicate via exosomes [61,62], and so our discovery that there is overlap in the control of these processes in SCs may also be relevant to these other systems.

It also remains to be investigated whether defective maturation of late endosomes and lysosomes, major sites for activation of the growth regulatory kinase complex, mechanistic Target of Rapamycin Complex 1 (mTORC1) [63,64], could underlie the inhibition of SC growth and membrane biogenesis observed after BMP signalling blockade. The potentially complex interplay between growth, trafficking and secretion makes it difficult to identify what changes are responsible for the defect in reprogramming female receptivity that accompanies BMP signalling inhibition in SCs [23]. Although we have previously provided evidence that this may be partly explained by altered exosome secretion [17], our findings here suggest that other secretory processes and a general reduction in macromolecular biosynthesis in SCs may also be involved.

Overall, we conclude that SCs match secretion of DCG compartments with their replenishment by regulating DCG biogenesis rates via BMP signalling induced by DCG-packaged Dpp. Evidence from other systems suggests that autocrine BMP signalling may provide a general mechanism for control of DCG biogenesis rates in response to regulated secretion. For example, neuronal Gbb synthesis is required for normal synaptic secretion at the neuromuscular junction [65]. Furthermore, pancreatic β-cell signalling by the Dpp orthologue, BMP4, has been implicated in regulating glucose-stimulated insulin secretion via the BMP receptor BMPR1A [14]. It will now be important to test whether this signalling is truly autocrine and whether it could also match DCG biogenesis to secretion, particularly given the significance of secretory mis-regulation in some forms of diabetes.

## Materials and Methods

### Fly stocks

The following fly strains were used: esgF/O^ts^ (*w; esg-GAL4 tub-GAL80*^*ts*^ *UAS-FLP/CyO; UAS-GFP*_*nls*_ *actin>FRT>CD2>FRT>GAL4/TM6* [66];*UAS-CD63-GFP* [29]; *Acp26Aa-GAL4* [31]; *dsx-GAL4* [67]: *spi-GAL4*; *spi*^*NP0261*^ [68]; *UAS-GFP-GPI* [39]; *tub-rab11-YFP* [38]; *UAS-Dad* [25]; *UAS-tkv*^*QD*^ [23]; *UAS-gbb-GFP* [34]: *tkv*^*CPTI002487*^ [27]: *dpp*^*blk*^-GAL4 [35]; *UAS-Dpp-GFP* [33]; *UAS-tRFP-Dpp* [37]. Multiple lines were obtained from the Bloomington, Vienna and Kyoto Stock Centres including the following UAS-RNAi lines [69,70], some of which have been previously employed in other studies: *dpp*, JF01090 (RNAi#1; [71]), HMS00011 (RNAi# 2): *gbb*, HMS01243 [72]: *tkv*, JF01485 [73], JF01486: *wit*, HMS02298, JF01969: *sax*, v46358 [72], JF03431, HMS00758: *punt*, JF02664 [74], GL00069, HMS01944: *Mad*, JF01263 [75], JF01264: *Med*, JF02218 [72, 73], GL01313: *Snap24*, JF03146, v48034.

### Genetics

To achieve adult SC-specific expression of transgenes and RNAi, flies carrying UAS-coupled transgenes were crossed to flies containing *tub-GAL80*^*ts*^, a *UAS-GFP* transgene, and a GAL4 driver (*esg*, *spi*, *dsx*) that is only expressed in SCs of the AG [17, 23]. Typically, the progeny of this cross were shifted to 28.5°C on eclosion to inhibit the temperature-sensitive GAL4 repressor, GAL80^ts^, thus driving transgene expression specifically in adults. Mosaic SC expression was achieved by a temperature shift of esgF/O^ts^ progeny to 28.5°C in 5-day-old adults, when some SCs have stopped expressing *esg*-GAL4, as previously described [23]. For pulse experiments, flies were incubated from eclosion at 25°C then shifted to 28.5°C for the expression pulse.

### Mating experiments

To analyse the effects of mating on BMP signalling activity in SCs, control male flies were mated to a single female at either 3 days or 6 days post-eclosion. Immediately after mating, the male flies were separated and the AGs were dissected in fix and stained as described below. In experiments where a GFP-GPI expression pulse was employed, matings were set up at different times, as described in the text.

### Immunostaining and imaging

Immunostaining were performed as previously reported in [17,23]. Samples were incubated overnight with primary antibodies (diluted in PBST [PBS with 0.3% Triton X-100]) at 4°C before washing in PBST for 6 × 10 min. Incubation with fluorescently conjugated donkey secondary antibodies was for 2 h at 22°C followed by a further six10-min washes in PBST. Stained glands were mounted in Vectashield with DAPI. The following primary antibodies were used: ANCE (1:1000) [16], Wit (1:10; Developmental Biology Hybridoma Bank [DSHB]), Phospho-Smad1/5 (1:100; Cell Signalling; 41D10), Fasciclin 3 (1:10; DSHB), GFP (1:1000; Abcam; #6556). Secondary antibodies were conjugated to Alexa Fluor 649 and Cy3 (both diluted 1:400; Jackson ImmunoResearch Laboratories, Inc.) and incubated for 2 hours with AGs in the dark. All samples were imaged on a Zeiss Axioplan 2 (LSM 510 Meta) scanning confocal microscope, using LSM 510 Meta software. Low magnification images were obtained using the x10 objective (0.45NA, Plan-APOCHROMAT), whilst high magnification images used the x63 objective (1.4NA, Oil DIC, Plan-APOCHROMAT).

### Live cell imaging

AGs were dissected in ice-cold PBS and transferred to ice-cold PBS containing Lysotracker Red DN-99 (1:1000; Invitrogen) for 5 min. They were then washed in ice-cold PBS for 5 minutes, mounted in PBS on a coverslip bridge and imaged at approximately 16°C using an LSM 510 Axioplan 2 scanning confocal microscope (Zeiss) as described above.

### Counting compartment numbers

SVs were classified as CD63-positive, non-acidic compartments with a diameter of >1 μm. DCGs were defined as non-acidic compartments with a compact dense core marked by GFP-GPI or ANCE-cross-reactivity, and >1 μm in diameter. MVBLs were CD63-GFP-positive (on limiting membrane), LysoTracker Red-positive and > 2 μm in diameter, as in Corrigan et al. (2014). The compartments within each SC were counted by scanning through the entire cell. Three SCs were analysed per gland and the mean compartment number calculated.

### Assaying exosome release by SCs

For exosome lumen counts, 3-day-old UAS-CD63-GFP; *dsx*-GAL4 males expressing an RNAi against a target gene together with paired controls were mated with at least two *w*^*1118*^ virgin females at 29°C overnight to expel as much of the luminal AG content as possible. These males were isolated overnight at 29°C to allow replenishment of AG content and then dissected and fixed as previously outlined. Exosome secretion was measured using the approach described in [17], in which luminal CD63-GFP fluorescent puncta (exosomes or exosome aggregates) within the central third of each gland were counted in three different z-planes (spaced by 5 μm and imaged at identical settings using 1.7x zoom with the 63x oil objective) using ImageJ.

### Nuclear size measurement

Measurements were performed as described in [23]. The two nuclei of an individual SC and 3 surrounding MCs were imaged and their areas measured using Axiovision software (Zeiss). Three SC/MC clusters were imaged per gland. The relative SC:MC nuclear size was calculated and the ratios averaged across a minimum of 6 glands.

### Measurement of largest MVBL in SCs

The largest MVBL was identified by scanning through SCs and then imaged in the plane where the diameter was at its greatest. All images were taken using the same confocal settings. The diameter of the MVBL compartment was calculated using ImageJ.

### Measurement of fluorescent GFP inside MVBL

All images were taken using the same confocal settings, ensuring that the signal associated with MVBLs was not saturated. The z-plane in which the largest MVBL was at its greatest diameter was selected and a binary calculation was used to measure the proportion of the area covered by fluorescent GFP using ImageJ.

### Western blot analysis

Male AGs expressing SC-specific GFP-GPI from eclosion onwards and *w*^*1118*^ controls were dissected in ice-cold PBS. To generate female samples, each male expressing GFP-GPI was mated to a single *w*^*1118*^ female. The whole reproductive tract with ovaries removed was subsequently dissected from these females and from controls in ice-cold PBS.

The AG and female reproductive tract lysates were then prepared as previously described in [17]. Briefly, dissected samples were pooled and homogenised using a motorised pellet pestle (Sigma-Aldrich) in 100μl of lysis buffer (Radioimmunoprecipitation assay buffer; Sigma-Aldrich) supplemented with anti-protease and anti-phosphatase cocktails (Sigma-Aldrich). The lysate was then centrifuged (13000 g; 5 min) and protein content in the supernatant quantified using a bicinchoninic acid assay kit (Thermo Fisher Scientific).

Blots were prepared as previously described [16,17]. Samples containing 2.5 μg homogenized protein (made up to 15 μl) were added to 5 μl of 4× sampling buffer (0.2 M Tris-HCl, pH 6.8, 12% SDS, 40% glycerol, 20% β-mercaptoethanol, and 0.008% bromophenol blue) and heated to 95°C for 5 min. Proteins were separated on 10% mini-Protean TGX precast gels (Bio-Rad Laboratories) for 30 min along with a prestained ladder and then transferred to a polyvinylidene fluoride membrane (Immobilon-P; EMD Millipore) at 100 V for 1 h. The membrane was blocked for 1 h in 5% skimmed milk in TBS with Tween 20 (TBST), then incubated with anti-GFP antibody at 1:500 (rabbit; ab6556; Abcam) in 5% milk in TBST overnight at 4°C. It was washed in TBST for 3 × 5 min, incubated at room temperature for 1 h with a HRP-conjugated anti–rabbit secondary antibody (W4018; Promega) at 1:20,000 in 5% milk in TBST, and then washed in TBST for 3 × 5 min. Antibody binding was detected using Clarity Chemiluminescent Substrate (Bio-Rad) and a ChemiDoc Touch Imaging System (Bio-Rad). Band intensities were measured using the Image Lab software (Bio-Rad).

### Statistical analyses

For all compartment counts, measurements of intracellular compartments and nuclear size quantifications, three SCs per gland were analysed from a minimum of 10 animals (6 for nuclear size), typically involving 2–3 independent experiments. For analysis of pMad antibody staining patterns, all SCs in each AG were counted. All data were analysed using GraphPad Prism. The Shapiro-Wilk test was used to test for normality. Since not all data in each experiment were normally distributed, a Mann-Whitney *U* test or Kruskal-Wallis test was typically employed. Error bars represent ±1 SEM.

## Supporting Information

S1 Fig BMP signalling controls SC growth. **A-F**. Images of SCs from 6-day-old control male flies (A) or flies expressing RNAis targeting either, *wit* (B), *sax* (C), *punt* (D), or *Med* (E) or expressing an activated form of the Tkv receptor (Tkv^ACT^) (F) under the control of esgF/O^ts^ after temperature shift at eclosion. Green and red arrows indicate SC and MC nuclei respectively. Glands were stained with DAPI (blue) to mark nuclei and an anti-Fas3 antibody (yellow) to mark cell boundaries. Genotypes for images are: *w; esg-GAL4 tub-GAL80*^*ts*^ *UAS-FLP; UAS-GFP*_*nls*_ *actin>FRT>CD2>FRT>GAL4* combined with no other transgene (A); *P[TRiP*.*HMS02298]attP2* (III) (B); *P[GD2546]v46358* (II) (C); *P[TRiP*.*JF02664]attP2* (III) (D); *P[TRiP*.*JF02218]attP2* (III) (E); *P[w*^*+*^ *UAS-Tkv*^*ACT*^*]* (III) (F). Scale bar is 20 μm.
(TIF)

S2 Fig BMP signalling controls SC growth. **A.** Graph showing the effects on SC growth when BMP signalling is reduced using a second independent RNAi or increased after expression of an activated form of Tkv (expression driven by esgF/O^ts^ in A-F). **B**. The absolute size of SC nuclei is reduced when BMP signalling is inhibited. **C**. In contrast, MC nuclei are not affected when SCs express different genes targeting the BMP pathway. **D.** Flies carrying UAS-transgenes used in this study do not affect the SC:MC nuclear size ratio in the absence of an SC-specific driver. **E.** Driving expression of Dad and Tkv^ACT^ in adult SCs for 6 days with esgF/O^ts^, *dsx*-GAL4 and *spi*-GAL4 produces similar effects on SC nuclear growth. **F.** The esgF/O^ts^, *dsx*-GAL4 UAS-*CD63-GFP* and *spi*-GAL4 UAS-*GFP-GPI* lines all produce SCs with equivalent nuclear size relative to MCs when expression is induced for 6 days immediately after eclosion. **G**, **H**. SC-specific knockdown of *wit* using the esgF/O^ts^ driver line eliminates anti-Wit antibody staining in SCs (H) compared to control (arrow; G). Glands were stained with DAPI (blue) to mark nuclei. Gain settings on confocal are increased in (H) to demonstrate absence of specific signal. Genotypes for images are: *w; esg-GAL4 tub-GAL80*^*ts*^ *UAS-FLP; UAS-GFP*_*nls*_ *actin>FRT>CD2>FRT>GAL4* combined with no other transgene (G) or *P[TRiP*.*HMS02298]attP2* (III) (H). ***P<0.001, Kruskal-Wallis test, n = 10. Scale bar for G, H is 10 μm.
(TIF)

S3 Fig BMP signalling in SCs controls numbers of non-acidic compartments, endolysosomal maturation, largest MVBL size and exosome secretion. **A.** SC from dissected AG of 6-day-old male expressing CD63-GFP throughout adulthood and stained with an anti-GFP antibody and DAPI. Note absence in one large compartment of fluorescent GFP signal, which is still detected with the antibody (arrow). **B-F**. SCs expressing no RNAi (B) or RNAis targeted against transcripts encoding *wit* (C), *sax* (D), *punt* (E) and *Med* (F), and stained with Lysotracker Red (red). **G**. The number of SVs is reduced by inhibiting BMP signalling, using a second independent RNAi, except after *sax* knockdown, but increased by an activated form of Tkv. **H**. Inhibition of BMP signalling induces a significant accumulation of fluorescent GFP inside the largest MVBL (arrows in **B**-**F**), indicating a disruption in endolysosomal maturation. **I.** In most cases, inhibiting BMP signalling reduces the size of the largest MVBL. **J**. The number of CD63-GFP-positive exosomes released from SCs is reduced when BMP signalling is decreased. **K.** Expressing Dad and Tkv^ACT^ in SCs using either the *dsx*-GAL4 or *spi*-GAL4 driver produces a similar effect on the size of the largest MVBL. Genotypes for images are: *w; UAS-CD63-GFP tub-GAL80*^*ts*^*; dsx-GAL4* combined with no other transgene (A, B); *P[TRiP*.*HMS02298]attP2* (III) (C); *P[GD2546]v46358* (II) (D); *P[TRiP*.*JF02664]attP2* (III) (E); *P[TRiP*.*JF02218]attP2* (III) (F). The *w; UAS-CD63-GFP tub-GAL80*^*ts*^*; dsx-GAL4* line was employed to produce data in G-J. * P<0.05, ** P<0.01 ***P<0.001, Kruskal-Wallis test, n = 10. Scale bar for A-F 10 μm.
(TIF)

S4 Fig Dpp controls adult SC growth. **A**. Image of SCs from 6-day-old male expressing Dpp-GFP under the control of esgF/O^ts^ after temperature shift at eclosion and stained for pMad (red), which is present in all SC nuclei (arrows). **B**. Mosaic clones of SCs using esgF/O^ts^ system to express Dpp-GFP in some SCs, but not others. Note the presence of high levels of nuclear pMad only in SC expressing Dpp-GFP (white arrow) and not in adjacent non-expressing cell, which has smaller nuclei (white dashed circle). **C**. Expression of Dpp-GFP in MCs leads to nuclear pMad accumulation only in SCs (arrows). Glands in A-C were stained with DAPI (blue) to mark nuclei. **D**. Absolute SC nuclear size changes significantly when *dpp* is either silenced or Dpp-GFP is overexpressed in SCs, but no obvious change is seen for *gbb* (expression driven by esgF/O^ts^ in D-H). **E**. The same treatments produce no significant change in absolute MC nuclear size. **F**. Expression of Dpp-GFP in MCs induces SC-specific nuclear growth, but there is no clear change if *dpp*-RNAi or *gbb*-RNAi is expressed in MCs using the MC-specific *Acp26Aa*-GAL4 driver. **G**. SCs expressing a second independent RNAi targeting *dpp* transcripts show reduced relative nuclear growth. **H.** The UAS-*dpp*-RNAi construct has no effect on SC nuclear size in the absence of a GAL4 driver. **I**. Bar chart shows number of large compartments per cell labelled by anti-ANCE, GFP-GPI *w; spi-GAL4 tub-GAL80*^*ts*^ *UAS-GFP-GPI* line) or Rab11-YFP (*tub-Rab11-YFP* line). Counts were made in independent experiments using 3-day-old virgin males and do not differ significantly. Genotypes for images are: *w; esg-GAL4 tub-GAL80*^*ts*^ *UAS-FLP; UAS-GFP*_*nls*_ *actin>FRT>CD2>FRT>GAL4/UAS-Dpp-GFP* (A and mosaic in B); *w Acp26Aa*-GAL4; *UAS-Dpp-GFP (III)* (C). ***P<0.001, Kruskal-Wallis test, except for G and H, where a Mann-Whitney *U* test was used. Scale bar is 20 μm.
(TIF)

S5 Fig Fluorescently tagged Dpp traffics to DCGs. **A.** Males aged for 3 days were dissected and fixed after a 24 h pulse of CD63-GFP and Dpp-RFP, and SCs imaged. Dpp-positive cores (white arrows) are observed inside CD63-GFP-positive compartments. Note the increase in SC nuclear size (green arrows) even after one day of Dpp-RFP expression. **B.** Dpp-GFP expressed in a one hour pulse in adult males previously aged for 3days at 25°C after eclosion is chased into a number of spherical structures (arrows) that are located in ANCE-positive (red; eg. arrow in merged image) DCGs. Genotypes for images are: *w UAS-Dpp-tagRFP; tub-GAL80*^*ts*^*; dsx-GAL4* (A); *w; tub-GAL80*^*ts*^*; dsx-GAL4/UAS-Dpp-GFP* (B, C) genotypes; Scale bar is 10μm.
(TIF)

S6 Fig Expression of CD63-GFP in adult SCs increases DCG and SV compartment number. **A**. SCs in esgF/O^ts^, *dsx*-GAL4 *UAS-CD63-GFP* and *spi*-GAL4 *UAS-GFP-GPI* 6-day-old males that express their respective transgenes from eclosion onwards (after *tub-GAL80*^*ts*^ inactivation) all have the same number of MVBLs. **B**. The diameter of the largest MVBL in SCs from 6-day-old males is increased compared to wild type cells in esgF/O^ts^, *esg-GAL4 tub-GAL80*^*ts*^ *UAS-GFP-GPI*, *spi*-GAL4 *tub-GAL80*^*ts*^ *UAS-GFP-GPI*, *esg-GAL4 tub-GAL80*^*ts*^ *UAS-CD63-GFP*, *dsx*-GAL4 *tub-GAL80*^*ts*^ *UAS-CD63-GFP*, and *spi*-GAL4 *tub-GAL80*^*ts*^ *UAS-CD63-GFP* males. **C, D.** SC from 6-day-old *spi*-GAL4 *UAS-GFP-GPI* (C) and *spi*-GAL4 *UAS-GFP-GPI UAS-CD63-GFP* (D) males stained with Lysotracker Red to mark acidic compartments. **E.** Such co-expression of CD63-GFP with GFP-GPI for 6 days following eclosion produces SCs with increased numbers of GFP-GPI-positive DCG compartments compared to controls. Genotypes for images are: *w; spi-GAL4 tub-GAL80*^*ts*^ *UAS-GFP-GPI* combined with no other transgene (C) or with *UAS-CD63-GFP* (D). * P<0.05, ** P<0.01 ***P<0.001, Kruskal-Wallis test for A and B, for E a Mann-Whitney *U* test was used. Scale bar for C, D is 10 μm.
(TIF)

S7 Fig BMP signalling and mating control number of SCs with nuclear pMad staining. **A-C**. SCs from 6-day-old virgin males expressing either Dad (B), activated Tkv (C) or no BMP signalling regulator (A) stained with pMad antibody, revealing different BMP signalling activities. pMad-positive nuclei are marked with arrows. **D**. Graph showing the proportion of SCs that contain pMad-positive nuclei in 3-day-old esgF/O^ts^ male virgins and males dissected immediately after mating. **E**. Western blot probed with anti-GFP antibody. AG extracts from either *w*^*1118*^ males, or virgin and mated *w; spi-GAL4 tub-GAL80*^*ts*^ *UAS-GFP-GPI* males were analysed. Females were either virgins or mated to *w*^*1118*^ males or SC>GFP-GPI-expressing males.). Genotypes for images are: *w; esg-GAL4 tub-GAL80*^*ts*^ *UAS-FLP; UAS-GFP*_*nls*_ *actin>FRT>CD2>FRT>GAL4* combined with no other transgene (A); *P[w*^*+*^ *UAS-Dad]* (II) (B); *P[w*^*+*^ *UAS- Tkv*^*ACT*^*]* (III) (C).***P<0.001, Mann-Whitney *U* test, n>15. Scale bar is 20 μm.
(TIF)

S8 Fig BMP signalling components in SCs regulate BMP pathway activation and DCG number. **A.** Graph showing the proportion of SCs that contain pMad-positive nuclei in 6-day-old esgF/O^ts^ virgin males expressing RNAis against different BMP signalling components after eclosion. **B.** Graph showing that the number of GFP-GPI-labelled DCGs in SCs from 6-day-old *w; spi-GAL4 tub-GAL80*^*ts*^ *UAS-GFP-GPI* virgin males is reduced when BMP signalling components other than *sax* are knocked down post-eclosion.
(TIF)
