# Supplementary figures and images for: Regulation of Dense-Core Granule Replenishment by Autocrine BMP Signalling in Drosophila Secondary Cells
Source: PLoS Genet. 2016 Oct 11;12(10):e1006366. doi: 10.1371/journal.pgen.1006366 (PMC5065122; doi:10.1371/journal.pgen.1006366)

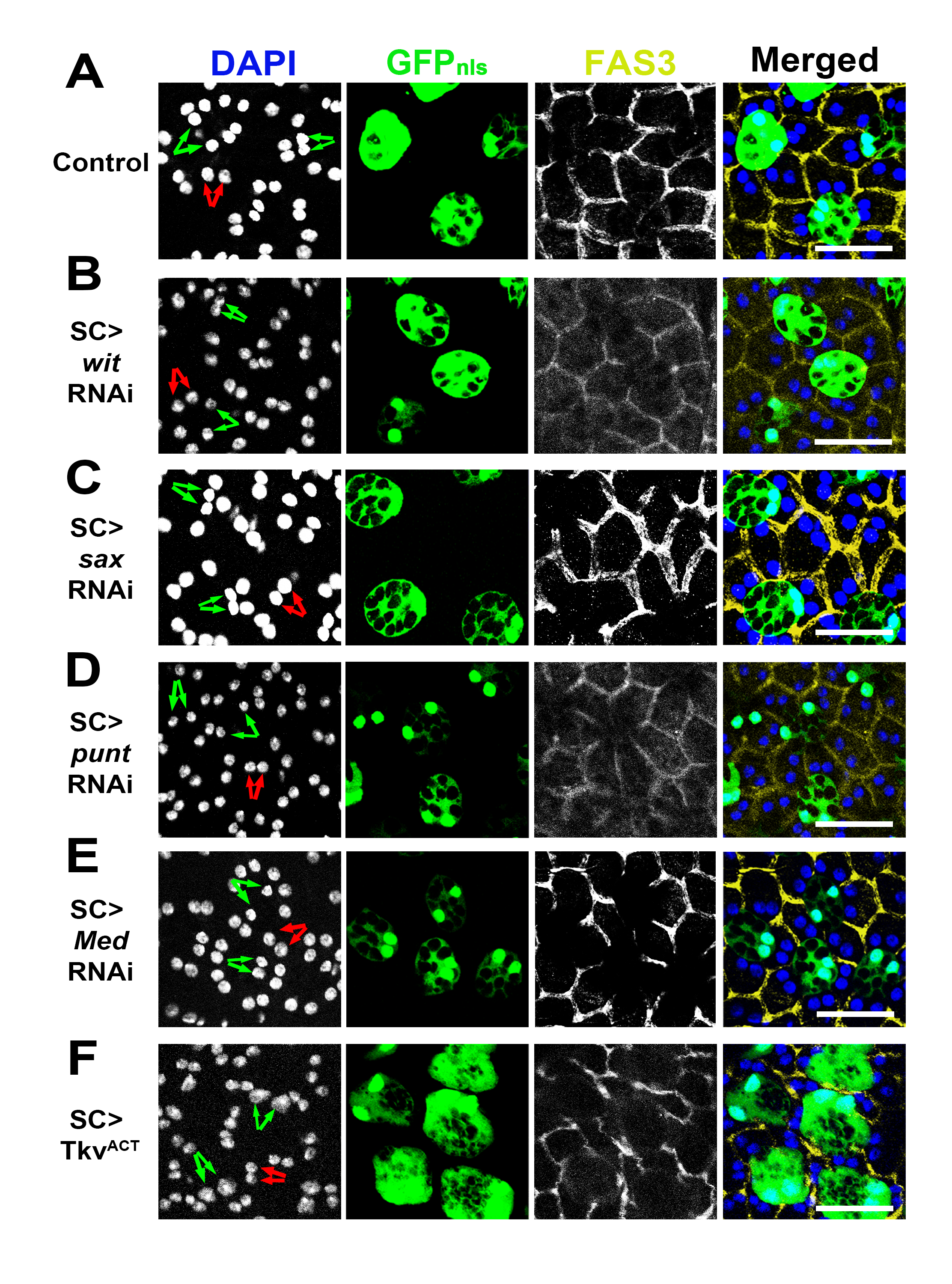

Supplement: S1 Fig — A-F. Images of SCs from 6-day-old control male flies (A) or flies expressing RNAis targeting either, wit (B), sax (C), punt (D), or Med (E) or expressing an activated form of the Tkv receptor (TkvACT) (F) under the control of esgF/Ots after temperature shift at eclosion. Green and red arrows indicate SC and MC nuclei respectively. Glands were stained with DAPI (blue) to mark nuclei and an anti-Fas3 antibody (yellow) to mark cell boundaries. Genotypes for images are: w; esg-GAL4 tub-GAL80ts UAS-FLP; UAS-GFPnls actin>FRT>CD2>FRT>GAL4 combined with no other transgene (A); P[TRiP.HMS02298]attP2 (III) (B); P[GD2546]v46358 (II) (C); P[TRiP.JF02664]attP2 (III) (D); P[TRiP.JF02218]attP2 (III) (E); P[w+ UAS-TkvACT] (III) (F). Scale bar is 20 μm. (TIF) [file pgen.1006366.s001.tif]

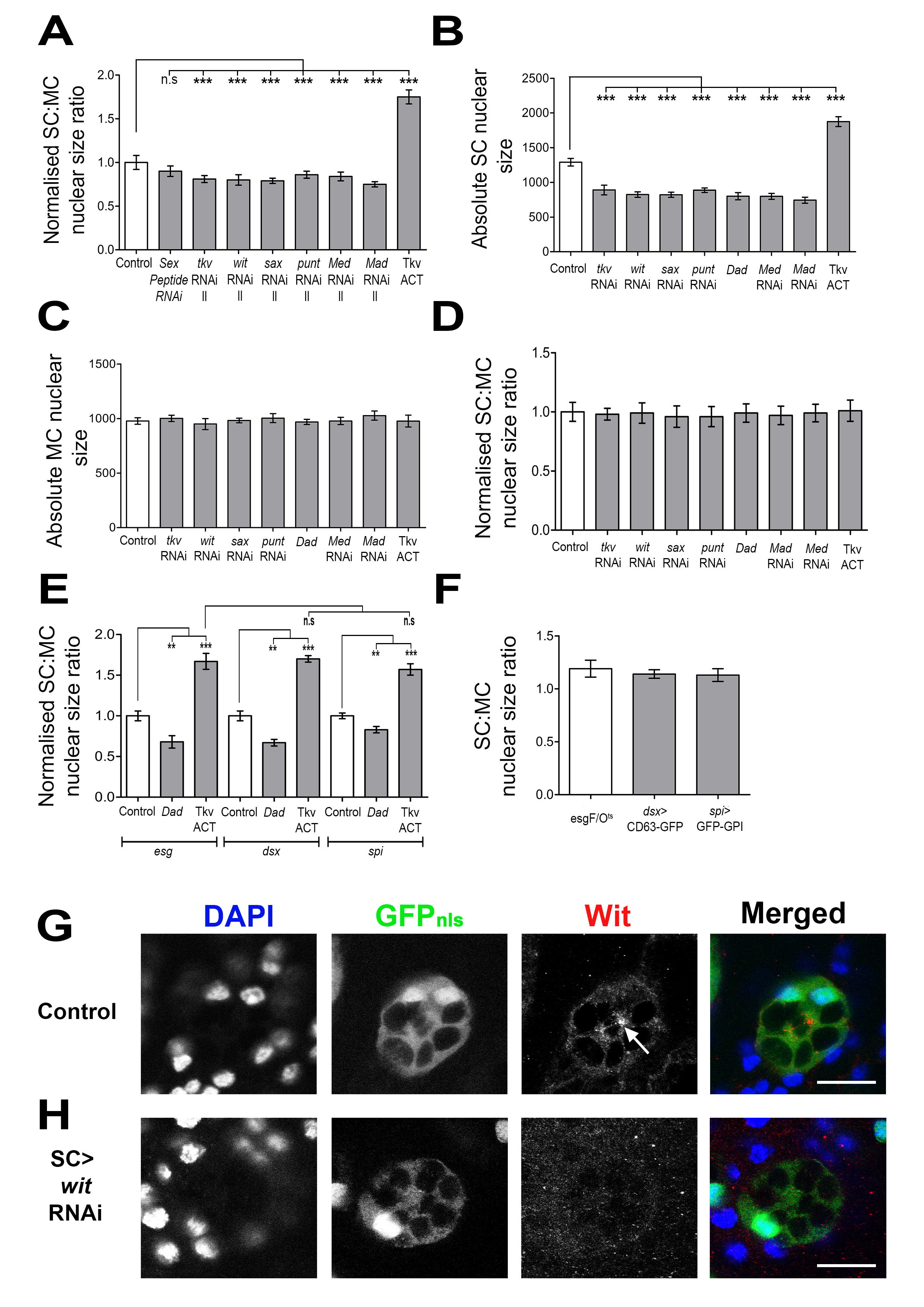

Supplement: S2 Fig — A. Graph showing the effects on SC growth when BMP signalling is reduced using a second independent RNAi or increased after expression of an activated form of Tkv (expression driven by esgF/Ots in A-F). B. The absolute size of SC nuclei is reduced when BMP signalling is inhibited. C. In contrast, MC nuclei are not affected when SCs express different genes targeting the BMP pathway. D. Flies carrying UAS-transgenes used in this study do not affect the SC:MC nuclear size ratio in the absence of an SC-specific driver. E. Driving expression of Dad and TkvACT in adult SCs for 6 days with esgF/Ots, dsx-GAL4 and spi-GAL4 produces similar effects on SC nuclear growth. F. The esgF/Ots, dsx-GAL4 UAS-CD63-GFP and spi-GAL4 UAS-GFP-GPI lines all produce SCs with equivalent nuclear size relative to MCs when expression is induced for 6 days immediately after eclosion. G, H. SC-specific knockdown of wit using the esgF/Ots driver line eliminates anti-Wit antibody staining in SCs (H) compared to control (arrow; G). Glands were stained with DAPI (blue) to mark nuclei. Gain settings on confocal are increased in (H) to demonstrate absence of specific signal. Genotypes for images are: w; esg-GAL4 tub-GAL80ts UAS-FLP; UAS-GFPnls actin>FRT>CD2>FRT>GAL4 combined with no other transgene (G) or P[TRiP.HMS02298]attP2 (III) (H). ***P<0.001, Kruskal-Wallis test, n = 10. Scale bar for G, H is 10 μm. (TIF) [file pgen.1006366.s002.tif]

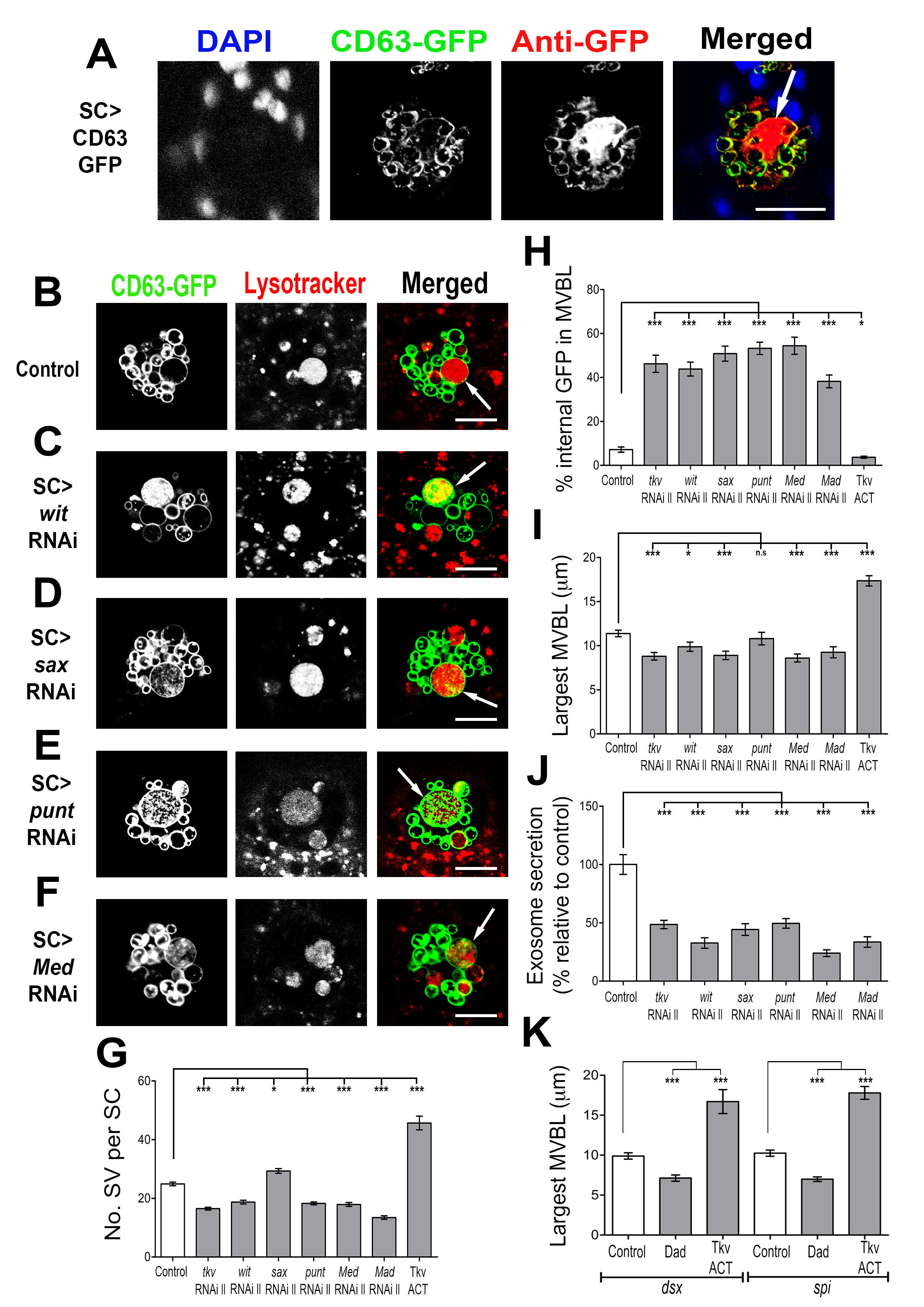

Supplement: S3 Fig — A. SC from dissected AG of 6-day-old male expressing CD63-GFP throughout adulthood and stained with an anti-GFP antibody and DAPI. Note absence in one large compartment of fluorescent GFP signal, which is still detected with the antibody (arrow). B-F. SCs expressing no RNAi (B) or RNAis targeted against transcripts encoding wit (C), sax (D), punt (E) and Med (F), and stained with Lysotracker Red (red). G. The number of SVs is reduced by inhibiting BMP signalling, using a second independent RNAi, except after sax knockdown, but increased by an activated form of Tkv. H. Inhibition of BMP signalling induces a significant accumulation of fluorescent GFP inside the largest MVBL (arrows in B-F), indicating a disruption in endolysosomal maturation. I. In most cases, inhibiting BMP signalling reduces the size of the largest MVBL. J. The number of CD63-GFP-positive exosomes released from SCs is reduced when BMP signalling is decreased. K. Expressing Dad and TkvACT in SCs using either the dsx-GAL4 or spi-GAL4 driver produces a similar effect on the size of the largest MVBL. Genotypes for images are: w; UAS-CD63-GFP tub-GAL80ts; dsx-GAL4 combined with no other transgene (A, B); P[TRiP.HMS02298]attP2 (III) (C); P[GD2546]v46358 (II) (D); P[TRiP.JF02664]attP2 (III) (E); P[TRiP.JF02218]attP2 (III) (F). The w; UAS-CD63-GFP tub-GAL80ts; dsx-GAL4 line was employed to produce data in G-J. * P<0.05, ** P<0.01 ***P<0.001, Kruskal-Wallis test, n = 10. Scale bar for A-F 10 μm. (TIF) [file pgen.1006366.s003.tif]

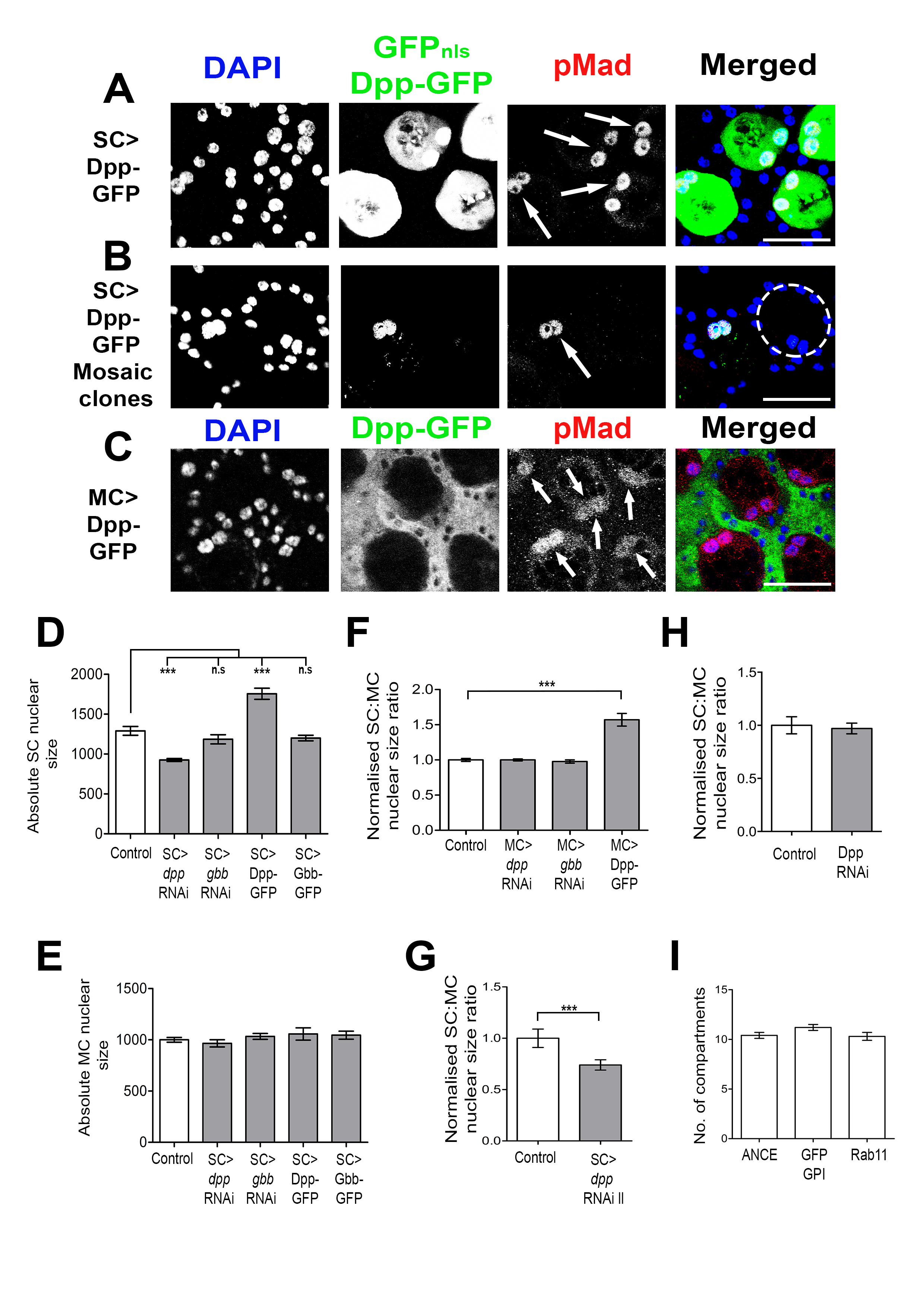

Supplement: S4 Fig — A. Image of SCs from 6-day-old male expressing Dpp-GFP under the control of esgF/Ots after temperature shift at eclosion and stained for pMad (red), which is present in all SC nuclei (arrows). B. Mosaic clones of SCs using esgF/Ots system to express Dpp-GFP in some SCs, but not others. Note the presence of high levels of nuclear pMad only in SC expressing Dpp-GFP (white arrow) and not in adjacent non-expressing cell, which has smaller nuclei (white dashed circle). C. Expression of Dpp-GFP in MCs leads to nuclear pMad accumulation only in SCs (arrows). Glands in A-C were stained with DAPI (blue) to mark nuclei. D. Absolute SC nuclear size changes significantly when dpp is either silenced or Dpp-GFP is overexpressed in SCs, but no obvious change is seen for gbb (expression driven by esgF/Ots in D-H). E. The same treatments produce no significant change in absolute MC nuclear size. F. Expression of Dpp-GFP in MCs induces SC-specific nuclear growth, but there is no clear change if dpp-RNAi or gbb-RNAi is expressed in MCs using the MC-specific Acp26Aa-GAL4 driver. G. SCs expressing a second independent RNAi targeting dpp transcripts show reduced relative nuclear growth. H. The UAS-dpp-RNAi construct has no effect on SC nuclear size in the absence of a GAL4 driver. I. Bar chart shows number of large compartments per cell labelled by anti-ANCE, GFP-GPI w; spi-GAL4 tub-GAL80ts UAS-GFP-GPI line) or Rab11-YFP (tub-Rab11-YFP line). Counts were made in independent experiments using 3-day-old virgin males and do not differ significantly. Genotypes for images are: w; esg-GAL4 tub-GAL80ts UAS-FLP; UAS-GFPnls actin>FRT>CD2>FRT>GAL4/UAS-Dpp-GFP (A and mosaic in B); w Acp26Aa-GAL4; UAS-Dpp-GFP (III) (C). ***P<0.001, Kruskal-Wallis test, except for G and H, where a Mann-Whitney U test was used. Scale bar is 20 μm. (TIF) [file pgen.1006366.s004.tif]

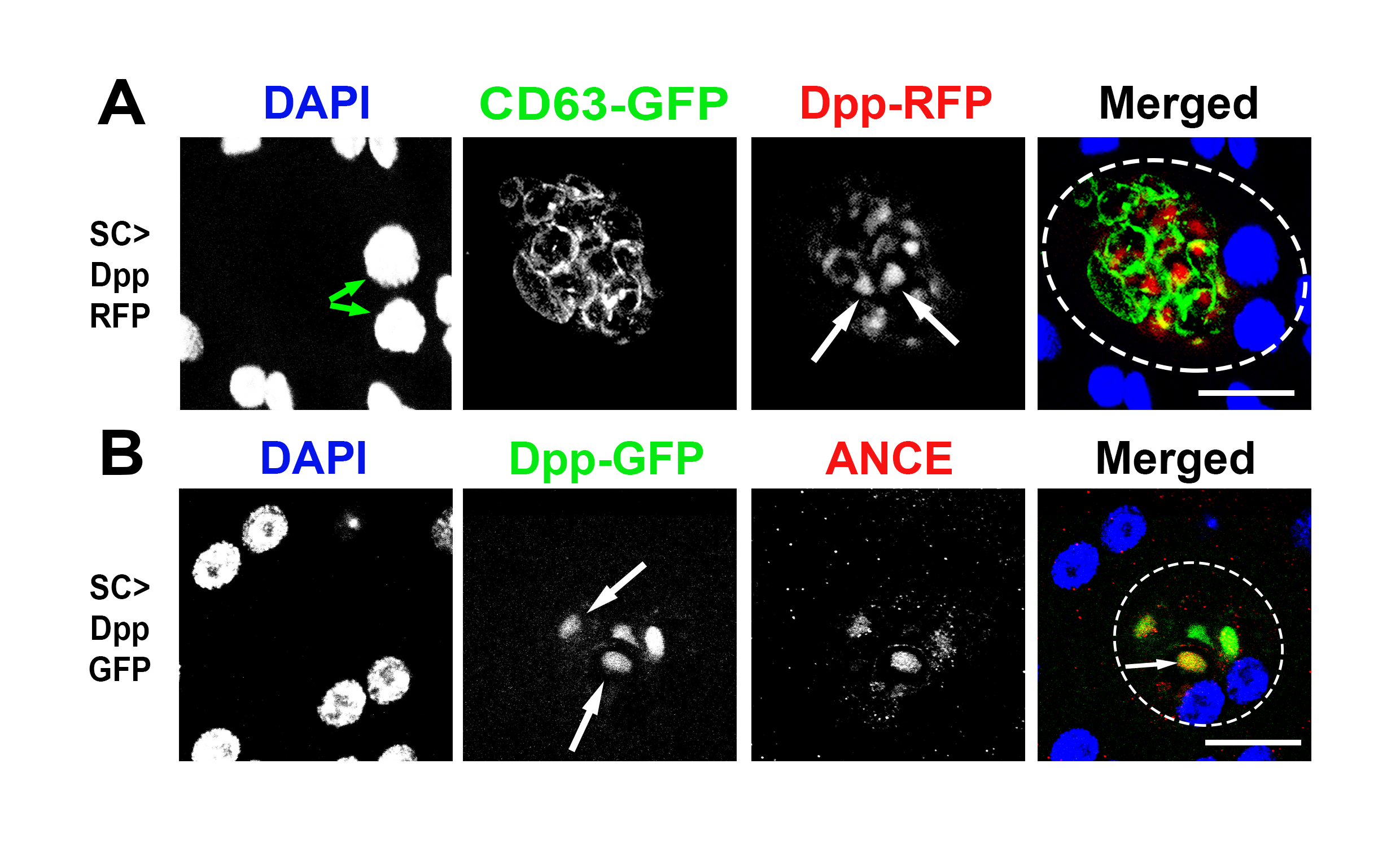

Supplement: S5 Fig — A. Males aged for 3 days were dissected and fixed after a 24 h pulse of CD63-GFP and Dpp-RFP, and SCs imaged. Dpp-positive cores (white arrows) are observed inside CD63-GFP-positive compartments. Note the increase in SC nuclear size (green arrows) even after one day of Dpp-RFP expression. B. Dpp-GFP expressed in a one hour pulse in adult males previously aged for 3days at 25°C after eclosion is chased into a number of spherical structures (arrows) that are located in ANCE-positive (red; eg. arrow in merged image) DCGs. Genotypes for images are: w UAS-Dpp-tagRFP; tub-GAL80ts; dsx-GAL4 (A); w; tub-GAL80ts; dsx-GAL4/UAS-Dpp-GFP (B, C) genotypes; Scale bar is 10μm. (TIF) [file pgen.1006366.s005.tif]

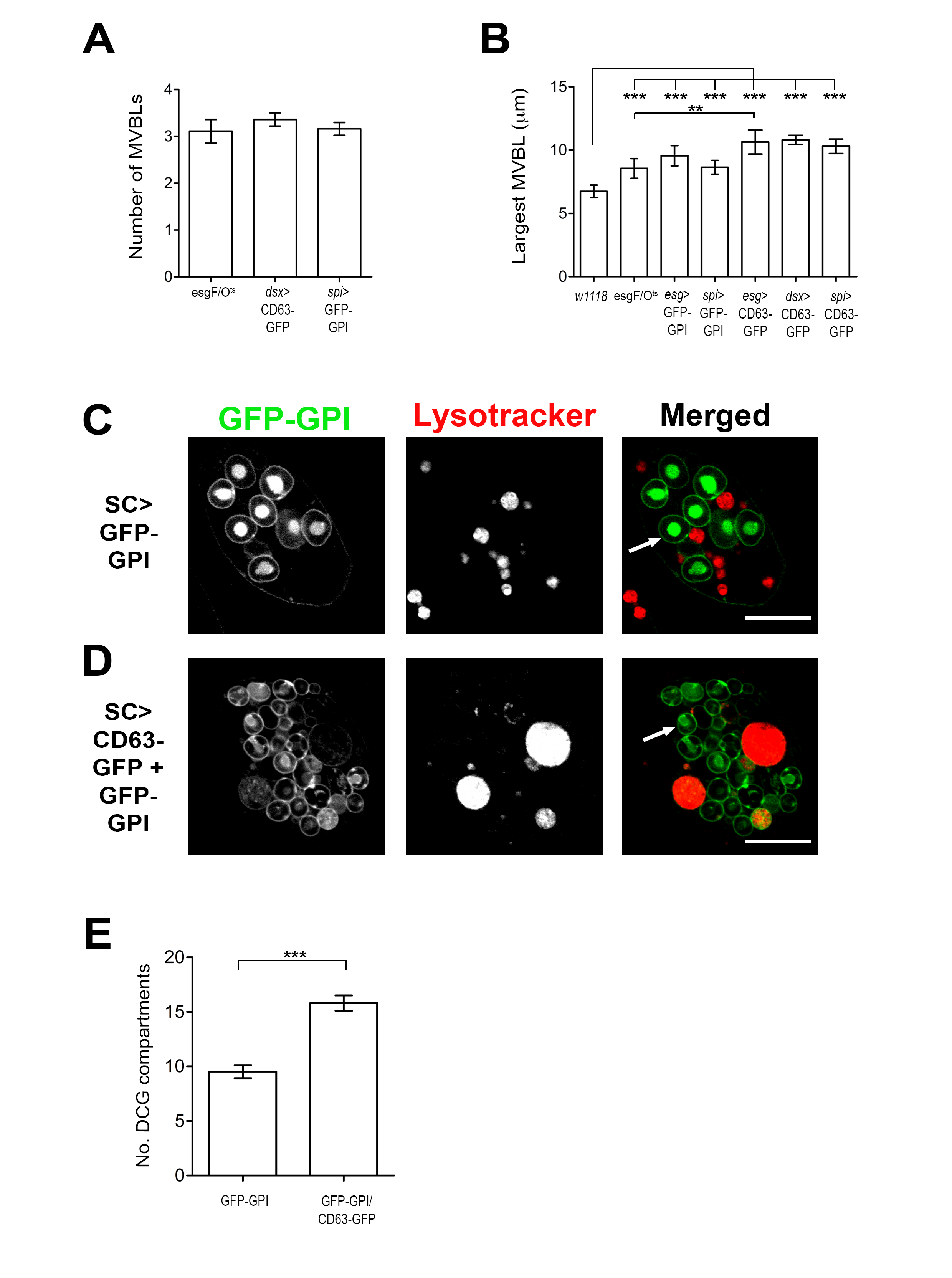

Supplement: S6 Fig — A. SCs in esgF/Ots, dsx-GAL4 UAS-CD63-GFP and spi-GAL4 UAS-GFP-GPI 6-day-old males that express their respective transgenes from eclosion onwards (after tub-GAL80ts inactivation) all have the same number of MVBLs. B. The diameter of the largest MVBL in SCs from 6-day-old males is increased compared to wild type cells in esgF/Ots, esg-GAL4 tub-GAL80ts UAS-GFP-GPI, spi-GAL4 tub-GAL80ts UAS-GFP-GPI, esg-GAL4 tub-GAL80ts UAS-CD63-GFP, dsx-GAL4 tub-GAL80ts UAS-CD63-GFP, and spi-GAL4 tub-GAL80ts UAS-CD63-GFP males. C, D. SC from 6-day-old spi-GAL4 UAS-GFP-GPI (C) and spi-GAL4 UAS-GFP-GPI UAS-CD63-GFP (D) males stained with Lysotracker Red to mark acidic compartments. E. Such co-expression of CD63-GFP with GFP-GPI for 6 days following eclosion produces SCs with increased numbers of GFP-GPI-positive DCG compartments compared to controls. Genotypes for images are: w; spi-GAL4 tub-GAL80ts UAS-GFP-GPI combined with no other transgene (C) or with UAS-CD63-GFP (D). * P<0.05, ** P<0.01 ***P<0.001, Kruskal-Wallis test for A and B, for E a Mann-Whitney U test was used. Scale bar for C, D is 10 μm. (TIF) [file pgen.1006366.s006.tif]

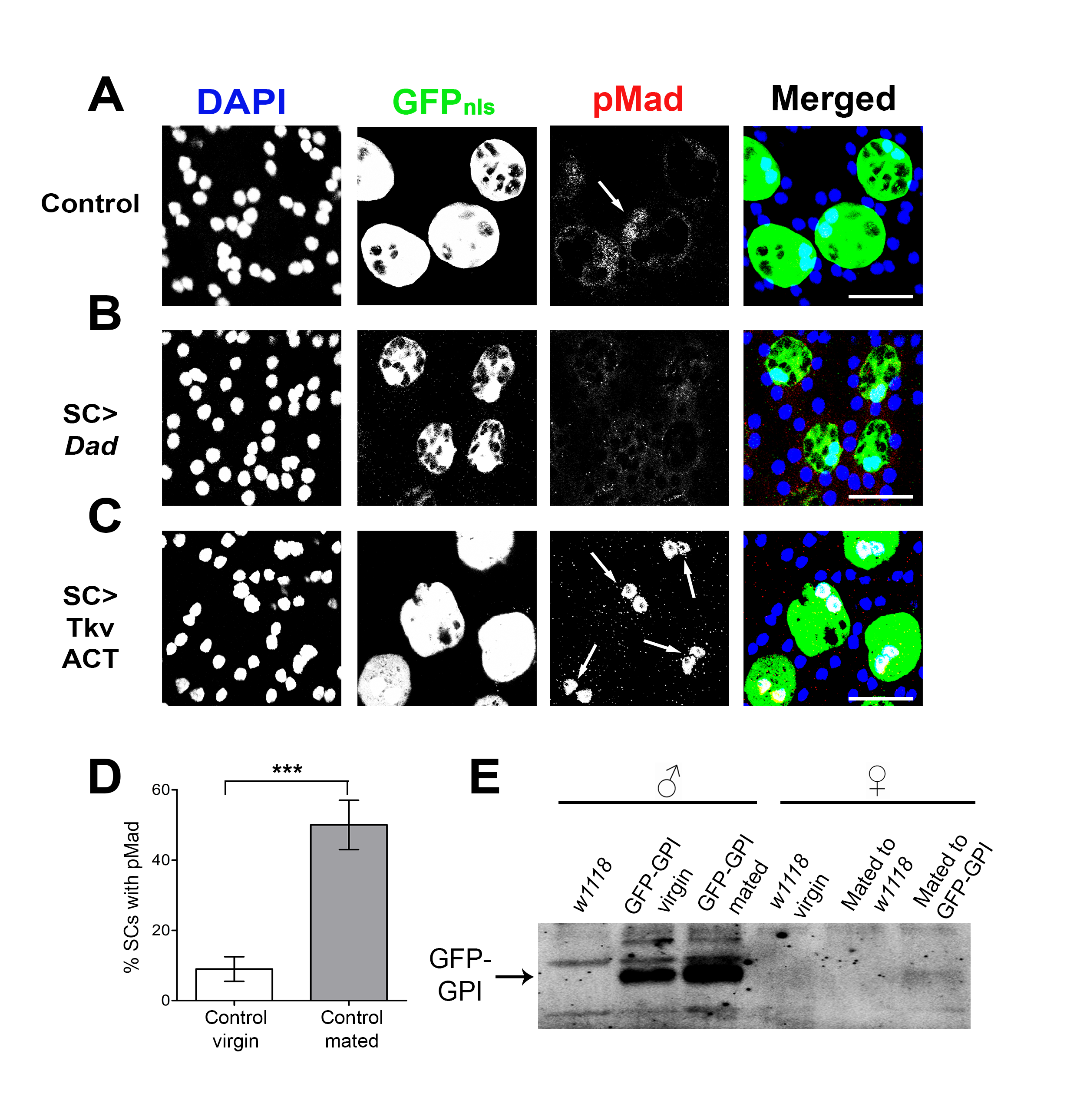

Supplement: S7 Fig — A-C. SCs from 6-day-old virgin males expressing either Dad (B), activated Tkv (C) or no BMP signalling regulator (A) stained with pMad antibody, revealing different BMP signalling activities. pMad-positive nuclei are marked with arrows. D. Graph showing the proportion of SCs that contain pMad-positive nuclei in 3-day-old esgF/Ots male virgins and males dissected immediately after mating. E. Western blot probed with anti-GFP antibody. AG extracts from either w1118 males, or virgin and mated w; spi-GAL4 tub-GAL80ts UAS-GFP-GPI males were analysed. Females were either virgins or mated to w1118 males or SC>GFP-GPI-expressing males.). Genotypes for images are: w; esg-GAL4 tub-GAL80ts UAS-FLP; UAS-GFPnls actin>FRT>CD2>FRT>GAL4 combined with no other transgene (A); P[w+ UAS-Dad] (II) (B); P[w+ UAS- TkvACT] (III) (C).***P<0.001, Mann-Whitney U test, n>15. Scale bar is 20 μm. (TIF) [file pgen.1006366.s007.tif]

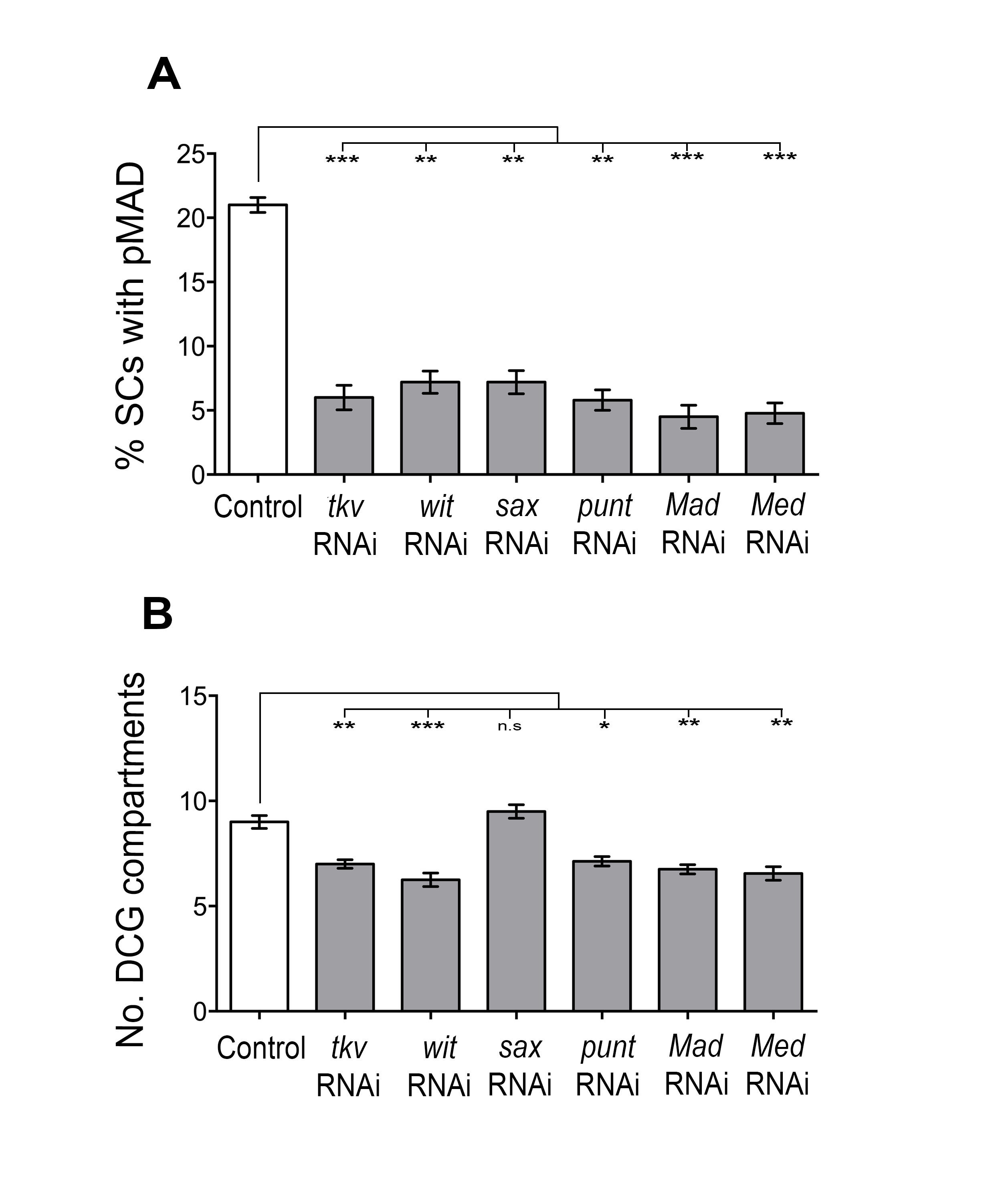

Supplement: S8 Fig — A. Graph showing the proportion of SCs that contain pMad-positive nuclei in 6-day-old esgF/Ots virgin males expressing RNAis against different BMP signalling components after eclosion. B. Graph showing that the number of GFP-GPI-labelled DCGs in SCs from 6-day-old w; spi-GAL4 tub-GAL80ts UAS-GFP-GPI virgin males is reduced when BMP signalling components other than sax are knocked down post-eclosion. (TIF) [file pgen.1006366.s008.tif]
